# Dispositional free riders do not free ride on punishment

**DOI:** 10.1038/s41467-018-04775-8

**Published:** 2018-06-19

**Authors:** Till O. Weber, Ori Weisel, Simon Gächter

**Affiliations:** 1 School of Economics, University College Dublin, Belfield, Dublin 4 Ireland; 2 Geary Institute for Public Policy, University College Dublin, Belfield, Dublin 4 Ireland; 3 Centre for Decision Research and Experimental Economics, Sir Clive Granger Building, University of Nottingham, University Park, Nottingham, NG7 2RD UK; 4 Coller School of Management, Tel Aviv University, 6997801 Tel Aviv, Israel; 5 CESifo, 81679 Munich, Germany; 6 IZA Institute of Labour Economics, 53113 Bonn, Germany

## Abstract

Strong reciprocity explains prosocial cooperation by the presence of individuals who incur costs to help those who helped them (‘strong positive reciprocity’) and to punish those who wronged them (‘strong negative reciprocity’). Theories of social preferences predict that in contrast to ‘strong reciprocators’, self-regarding people cooperate and punish only if there are sufficient future benefits. Here, we test this prediction in a two-stage design. First, participants are classified according to their disposition towards strong positive reciprocity as either dispositional conditional cooperators (DCC) or dispositional free riders (DFR). Participants then play a one-shot public goods game, either with or without punishment. As expected, DFR cooperate only when punishment is possible, whereas DCC cooperate without punishment. Surprisingly, dispositions towards strong positive reciprocity are unrelated to strong negative reciprocity: punishment by DCC and DFR is practically identical. The ‘burden of cooperation’ is thus carried by a larger set of individuals than previously assumed.

## Abstract (web summary)

Strong positive and strong negative reciprocators reward cooperation and punish defection, respectively, regardless of future benefits. Here, Weber and colleagues demonstrate that dispositions towards strong positive and strong negative reciprocity are not correlated within individuals.

## Introduction

The neoclassical Homo economicus model of humans as rational beings in narrow pursuit of their own self-interest does not sit well with the varied range of uniquely human phenomena that depend on self-sacrificial cooperative behaviour. Risk taking in large-scale conflict, caring for the sick and disabled, and periodic collective efforts, such as democratic movements combating authoritarian regimes or workers engaging in collective action, illustrate how humans stand out among other (cooperative) species by cooperating in large groups that extend genealogical kinship even when cooperation entails the forgoing of private welfare in the interest of the greater good^1–3^. Not everyone is a cooperator, though: some people are predisposed to cooperate, even at a cost, as long as they expect their group members to cooperate as well. Others never cooperate, irrespective of what they expect other group members to do. In the economics literature, these two types are commonly referred to as ‘conditional cooperators’ and ‘free riders’, respectively^4^. Cooperation in a population consisting of conditional cooperators and free riders is likely to deteriorate over time, because conditional cooperators’ willingness to cooperate would vanish in light of free riders’ reluctance to do so^5^.

A central result in the literature is that cooperation can be sustained through ‘strong reciprocity’, which entails that even in the absence of material incentives, e.g. in one-shot interactions with strangers, there is not only a willingness to pay a cost for cooperating with cooperative others (‘strong positive reciprocity’), but also for punishing non-cooperative others (‘strong negative reciprocity’)^6–13^. Thus, strong reciprocity is distinct from weak reciprocity, which refers to settings where cooperation and punishment can be rationalised by selfish, strategic incentives^1^.

In this paper, we test the hypothesis that strong positive and strong negative reciprocity are correlated at the individual level. Such a correlation implies that conditional cooperators are more likely than free riders to contribute to public goods in one-shot settings without punishment; and to punish non-contributors in one-shot settings with punishment^14,15^. The intuition for assuming this correlation is that costly punishment of non-cooperators can reasonably be viewed a form of second-order cooperation in itself^16–19^. Therefore, it is plausible to expect that individuals who refuse to engage in self-sacrificing first-order cooperation (‘free riders’) would be unwilling to spend resources for engaging in second-order cooperation in the form of punishment, and would rather free ride on the punishment of others.

More formally, social preference models in economics imply a correlation between strong positive and negative reciprocity by formalising reciprocity as unidimensional, using a single game-independent individual-level parameter to simultaneously capture preferences for both positive and negative reciprocity^20–22^. In these models, only people for whom this parameter is positive consider cooperating in a one-shot public goods game without punishment, or punishing when this opportunity exists; if the parameter equals zero, people care only about their own monetary payoff and never cooperate nor punish without strategic incentives for doing so. Similarly, models of inequality aversion^23,24^ assume a game-independent (lack of) aversion to inequality. Just like agents with no inclination towards reciprocity, agents who are not inequality averse, but are only motivated by material self-interest, will never cooperate (unless there is a threat of being punished) nor punish. Thus, from the viewpoint of prominent theories of social preferences, only people who are sufficiently motivated by inequality aversion or reciprocity will cooperate in the absence of punishment (strong positive reciprocity), and punish non-contributors in the presence of punishment (strong negative reciprocity).

The assumption of correlated strong positive and negative reciprocity is also present in theoretical and simulation-based work on cooperation in evolutionary biology. In these models, the population consists of (a) self-regarding agents who neither cooperate with cooperative others nor punish non-cooperative others, (b) conditional cooperators who are willing to cooperate, but do not punish, and (c) strong reciprocators who both cooperate and punish. The fourth logically possible type—punishers who do not cooperate—is explicitly omitted (some models also omit cooperators)^7,25,26^.

Lastly, the hypothesis that strong positive and negative reciprocity are correlated is in line with a common pattern in the experimental literature on cooperation and punishment, whereby agents who cooperate are also those who tend to punish free riders^14,27–32^. This pattern, however, may be misleading, due to the typical setup of most of these experiments. With some exceptions^33–35^, dispositions towards cooperation (i.e. whether individuals are strong positive reciprocators or not) are not measured independently, but are inferred from behaviour in settings where players’ moves are simultaneous, their payoffs are interdependent, and there is a threat of being punished. While the finding that cooperators constitute the majority of punishers in such settings does not contradict the hypothesis that strong positive and negative reciprocity are correlated, it is also not convincing support of it, and certainly not proof, because cooperation is not influenced only by dispositions towards cooperation, but also by beliefs about the cooperation and punishment of others. To illustrate, consider an individual who is in principle willing to positively reciprocate others’ cooperation, but happens to be pessimistic about their actual cooperation. This person may refrain from cooperation, despite her positive reciprocal tendency, behaving as a free rider who will not cooperate even if others do. Similarly, cooperation when there is a threat of punishment is not necessarily an indication of a disposition to cooperate, but might be a result of the changed incentives (i.e. fear of being punished).

Is the correlation between strong positive and negative reciprocity necessary, in principle, to explain cooperation? The answer seems to be ‘no’, according to models showing that punishment can sustain cooperation without requiring conditional cooperation at all^36^, and, more generally, that dispositions towards seemingly irrational behaviours—in our case, either costly cooperation or costly punishment—can emerge, independently of each other, as an effective way to solve commitment problems^37^. Given the basic maxim of strong reciprocity—that, in addition to strong positive reciprocity, strong negative reciprocity is also required to explain cooperation—the reliance on strong reciprocators to both cooperate with cooperative others and to punish uncooperative others, or even the existence of such a group of individuals, is not a logical necessity. As long as punishment provides sufficient incentives to steer free riders towards cooperation, the identity of the punishers is inconsequential; it should not matter if the punishers are predisposed towards cooperating with cooperative others (dispositional conditional cooperators (DCC)) or not (dispositional free riders (DFR)).

As explained above, existing evidence in seeming support of the hypothesis of a positive correlation of strong positive and negative reciprocity—and also against it^38–42^—does not, with exceptions^33–35^, control for beliefs and expectations about others’ behaviour when determining dispositions towards cooperation. The current work makes a distinction between agents’ intrinsic disposition towards (first-order) cooperation and their actual cooperative behaviour in the presence of punishment, by employing a two-phase experimental design. In the first phase, we classify participants according to their disposition towards strong positive reciprocity as either ‘Dispositional Conditional Cooperators’ (strong positive reciprocators; DCC) or ‘Dispositional Free Riders’ (not willing to positively reciprocate others’ cooperation; DFR). In the second phase, we examine their cooperation and punishment behaviour (which, to be clear, can differ from their disposition) in a one-shot public goods game either with or without punishment (study 1), or with different levels of punishment effectiveness (study 2). Importantly, in both studies, the classification procedure in the first phase controls for beliefs about the behaviour of others and is thus a clean measure of dispositions towards strong positive reciprocity. The second phase, a one-shot interaction in which there is no material incentive to use punishment, is a clean measure of dispositions towards strong negative reciprocity (see Methods).

The main predictions that arise from our reading of the literature are that (a) only DCC contribute to the public good in the absence of punishment; (b) to avoid punishment, DFR, as well as DCC, contribute to the public good when punishment is available^15^; and (c) only DCC will spend resources to punish non-cooperators. Strong negative reciprocity acts (punishment) are predicted to be exerted only by DCC, who by definition are strong positive reciprocators.

We also investigate the role of emotions in the decision making of DCC and DFR. Negative emotions—particularly anger—have been shown to be a proximate mechanism behind strong negative reciprocity^14,43–45^. The punishment of non-cooperators serves as an outlet for negative emotions, and the psychological reward associated with punishment can outweigh the material cost^46^. We utilise the link between negative emotions and costly punishment to explore potential individual differences in the proximate explanation of punishment. If DFR never punish, the question arises: do DFR experience less anger than DCC in the face of others’ defection, or do DFR experience similar levels of anger, but still refrain from punishing? To address these questions, and, if DFR do engage in punishment, to understand whether they do so for similar reasons to DCC, we elicit the intensity of a range of emotions experienced by participants.

Our results support the first two hypotheses, but refute the third. DCC are indeed much more willing than DFR to cooperate in the absence of punishment. When there is a threat of punishment, DFR increase their contributions to nearly DCC-like levels. Surprisingly and in contrast to the third hypothesis, DCC and DFR are highly similar in the way they use punishment, with comparable levels of punishment, motives for punishment, and emotional response to non-cooperators. In other words, DFR—as compared to DCC—do not free ride on others’ costly punishment of non-cooperators.

## Results

### Dispositions towards strong positive reciprocity (study 1)

Following the first phase, 48.9% of the players were classified as DCC, whose contribution schedule is increasing in the contributions of their group members, and 26.6% of the players as DFR, who are not willing to contribute anything regardless of the contribution of the other group members. The remaining 24.5% are unclassified and excluded from analysis (see Methods and Supplementary Notes 1, 2 for the classification criteria and additional details).

### Beliefs and contribution behaviour (study 1)

**Fig. 1:**
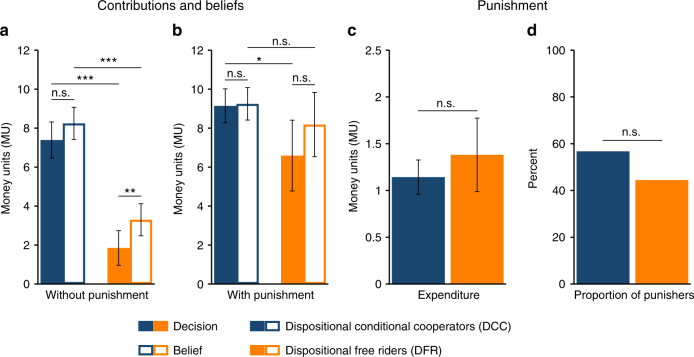
Contribution and prosocial punishment behaviour of DCC and DFR. **a** Contributions and beliefs in the without punishment treatment (*N*_DCC_ = 41; *N*_DFR_ = 27). Asterisks refer to Mann–Whitney/Wilcoxon signed-rank tests; n.s. *P* ≥ 0.10; **P < 0.05; ***P < 0.01. **b** Contributions and beliefs in the with punishment treatment (*N*_DCC_ = 49; *N*_DFR_ = 22). Asterisks refer to Mann–Whitney/Wilcoxon signed-rank tests; n.s. *P* ≥ 0.10; *P < 0.10; ***P < 0.01. **c** Average expenditure on prosocial punishment. n.s. *P* > 0.10; Mann–Whitney test. **d** Proportion of punishers engaged in prosocial punishment. n.s. *P* > 0.10; *χ*^2^ test. The error bars indicate ± 1 SEM

Without punishment both DCC and DFR behave in a way that is true to their disposition towards cooperation (Fig. 1a). A full 78% of DFR are perfectly consistent with their elicited disposition and contribute nothing, whereas 76% of DCC make a positive contribution (*M*_DFR_ = 1.85, *M*_DCC_ = 7.39, Mann–Whitney *z* = 4.23, *P* < 0.001). Both DCC and DFR contribute less than what they believe others to contribute, but the difference is significant only for DFR (DCC: *M*_contribution_ = 7.39, *M*_belief_ = 8.24, Wilcoxon signed-rank test *z* = −1.08, *P* = 0.282; DFR: *M*_contribution_ = 1.85, *M*_belief_ = 3.30, *z* = −2.22, *P* = 0.027). Interestingly, beliefs of DFR are significantly lower than beliefs of DCC (*M*_DFR_ = 3.30, *M*_DCC_ = 8.24, Mann–Whitney *z* = 3.93, *P* < 0.001).

The presence of punishment opportunities (Fig. 1b) has little effect on DCC, with neither beliefs nor contributions significantly differing as compared to without punishment (Mann–Whitney *z*_belief_ = −0.83, *P*_belief_ = 0.409; *z*_contribution_ = −1.34, *P*_contribution_ = 0.180). Beliefs and behaviour of DFR, however, are dramatically affected by punishment (Mann–Whitney *z*_belief_ = −2.00, *P*_belief_ = 0.046; *z*_contribution_ = −2.00, *P*_contribution_ = 0.045), increasing to DCC-like levels. Note that this shift in beliefs and contributions does not necessarily reflect a change in DFR’s disposition towards cooperation; rather, it is likely to reflect the expectation that the opportunity to punish will raise overall contributions, and a desire to avoid being punished by contributing (almost) as much as the others.

Overall, the results with respect to beliefs and contributions are consistent with strong positive reciprocity. Without punishment, DCC positively reciprocate what they expect others to contribute, conditioning their own contribution on their beliefs about others’ contributions, while DFR mostly refrain from contribution.

### Punishment behaviour (study 1)

Our hypotheses postulate that only DCC will bear the cost of punishing defectors. In the absence of material incentives, as is the case in the one-shot game that we consider, DFR are expected to never invest resources to punish others. In sharp contrast, the average investment in punishment that is directed at defectors is higher for DFR than for DCC (albeit not significantly different; *M*_DFR_ = 1.38, *M*_DCC_ = 1.14; Mann–Whitney *z* = 0.32, *P* = 0.749; each participant as an independent observation for all tests in this section; Fig. 1c), and DFR are not significantly different from DCC in the share of participants who use punishment when encountering a group member who contributed less than themselves (57% vs. 44%; *χ*^2^(1) = 0.44, *P* = 0.506, *N* = 46; Fig. 1d). Additional to the punishment of defectors shown in Fig. 1, sanctioning cooperators (i.e. antisocial punishment^30,31,47–49^) was also permitted. However, engagement in antisocial punishment is generally low and differences between DCC and DFR are not significant (Supplementary Note 3).

We use a regression analysis to control for factors that can potentially influence the punishment decision (Supplementary Note 4). The results confirm our findings reported above. Both the frequency and severity of punishment are not significantly different between DCC and DFR. The only difference in the punishment behaviour of DCC and DFR is an opposing peer effect: as other group members contribute more, DCC are more likely to punish a defector, but DFR are less likely to punish a defector. A possible explanation is that DCC perceive others’ high contributions as a signal for a high contribution norm, which makes them more likely to enforce this norm^50^. The regression results are robust to a stricter classification of DCC, as well as to a continuous measure of strong positive reciprocity.

### Emotions (study 1)

**Fig. 2:**
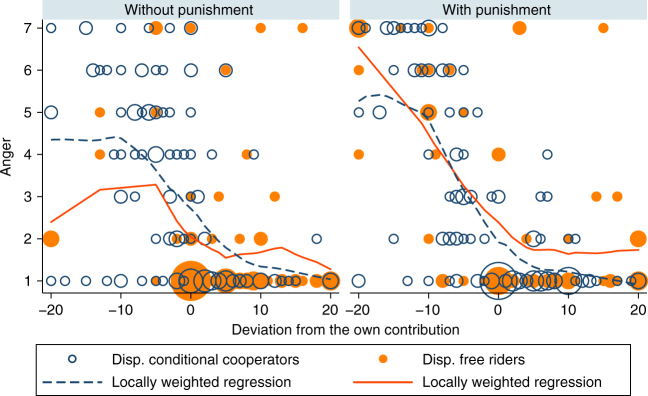
Self-reported anger levels depend on the deviation from the own contribution. Participants indicated the intensity of anger on a scale from 1 (not at all) to 7 (very much). The size of the bubbles corresponds to the number of observations at this location. The lines indicate the locally weighted regression functions of DCC (dark blue) and DFR (orange), and are very similar for both types. In both treatments, DCC and DFR feel angrier as group member’s negative deviations from the own contribution are higher

In line with previous studies^51,52^, we find a strong link between punishment expenditure and the intensity of negative emotions one experiences (Supplementary Note 5). All five negative emotions included in the questionnaire (anger, contempt, envy, irritation and jealousy) are positively correlated with punishment expenditure. In the following we focus on anger, the central moral emotion connected with norm transgressions^53^. Figure 2 illustrates the self-reported anger of DCC and DFR depending on the degree to which others’ contribution deviates from their own contribution. In both the without punishment and with punishment treatments, DCC and DFR exhibit higher anger levels as the negative deviation of others’ increases, and in both treatments DCC and DFR mostly feel no anger towards group members who contributed at least as much as they did. A regression analysis shows no significant level differences between DCC and DFR in the with punishment treatment (Supplementary Note 5). The only difference between DCC and DFR is that DFR are significantly angrier than DCC when a group member contributed more than them. Overall, DCC and DFR are highly similar in the way anger is related to the behaviour of others.

### Summary of study 1

The aim of study 1 was to provide a direct test of the often-invoked assumption that strong positive reciprocity (conditional cooperation) and strong negative reciprocity (punishment) are linked: strong reciprocators are necessarily DCC and may punish if others contribute less than them; DFR never punish, and cooperate only if there is a threat of punishment. The main result of study 1 is that the individual tendency to punish defectors is independent of dispositions towards strong positive reciprocity: the punishment behaviour of DCC and DFR is virtually indistinguishable; both punish those who contribute less than them to a similar degree. This result is reminiscent of ‘selfish punisher’ types who do not contribute to the public good, but punish other non-contributors^54,55^. The difference is that in our experiment DFR do make positive contributions to the public good when there is a threat of punishment and then, like DCC, also punish those who contributed less than them. In fact, DFR are not only similar to DCC in their punishment behaviour, but also increase their contributions to the public good when facing the threat of punishment.

### Motives to punish

Even if their punishment behaviour is highly similar, DCC and DFR might have different motives to punish in study 1. Given the 2:1 punishment ratio in study 1, (prosocial) punishment harms the punished person, but also reduces the absolute payoff differences between the punisher and the punished person. Study 2 is designed to separate these two motivations by changing the punishment ratio such that punishment no longer reduces payoff differences, while keeping contribution levels similar to those in study 1.

Study 2 closely follows the two-phase design of the with punishment treatment in study 1. The first phase was identical to that of study 1. The second phase was different; it included two punishment conditions that differ in the punishment ratio^27,56–59^ (see Methods). In one condition, the ratio was 3:1, which allowed the punisher to reduce the absolute payoff difference vis-à-vis a punished free rider. Crucially, in the other condition the punishment ratio was 1:1, which did not allow the punisher to change the absolute difference in payoffs between herself and the punished person, thus excluding one of the motives for prosocial punishment present in study 1.

A novel feature of study 2 is that the actual punishment ratio applicable to each participant was determined—by an individual random draw—only after making the contribution decisions in the second phase. Each participant had a 50% chance of drawing the 3:1 punishment ratio (for each MU this person spent on punishment, the punished group member lost 3 MU), and a 50% chance of drawing the 1:1 punishment ratio (for each MU this person spent on punishment, the punished group member lost 1 MU). This procedure has two important advantages: (a) contribution levels and beliefs about others’ contributions were kept constant across the two conditions, allowing for a clean comparison of punishing behaviour, as everything preceding the punishment decision is identical; (b) the expected payoff reduction of each MU spent on punishment was 2 MU, as in study 1, allowing for a comparison between the studies. We recruited 272 participants to take part in study 2; 135 were randomly assigned to the 3:1 punishment ratio condition, and 137 to the 1:1 punishment ratio condition.

### Dispositions towards strong positive reciprocity (study 2)

Following the first phase, 55.5% of participants were classified as DCC, 27.9% as DFR, and 16.5% remained unclassified (and were excluded from the analysis). The distribution of types in study 2 is very similar to that of study 1, *χ*^2^(2) = 4.46, *P* = 0.108, *N* = 456.

### Beliefs and contribution behaviour (study 2)

Contributions in study 2 (*M* *=* 9.62, SD = 7.12), as well as beliefs about others’ contributions (*M* *=* 9.72, SD = 6.01), were similar to those in study 1 (Mann–Whitney *z* = −1.31, *P* = 0.191; *z* = −0.97, *P* = 0.333; resp.), confirming that our novel punishment procedure indeed created similar incentives to contribute as compared to the with punishment treatment in study 1 (Supplementary Note 6).

### Punishment behaviour (study 2)

Pooling the 3:1 and 1:1 punishment ratio conditions in study 2, we find that the novel punishment procedure had little effect on overall prosocial punishment expenditures, with both DCC and DFR spending similarly in study 1 and 2 (Mann–Whitney *z*_DCC_ = 1.11, *P*_DCC_ = 0.267; *z*_DFR_ = 0.57, *P*_DFR_ = 0.572; each participant as an independent observation for all tests in this section). See Supplementary Note 7 for additional details.

**Fig. 3:**
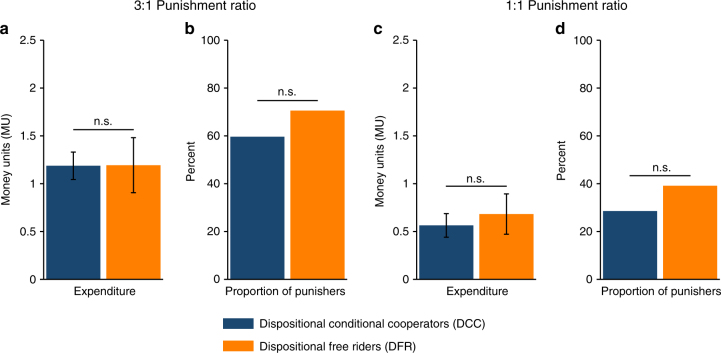
Prosocial punishment behaviour of DCC and DFR in the 3:1 punishment ratio condition and the 1:1 punishment ratio condition. **a** Average expenditure of DCC and DFR on prosocial punishment in the 3:1 punishment ratio condition (*N*_DCC_ = 77; *N*_DFR_ = 35); Mann–Whitney test, n.s. *P* > 0.10. **b** Share of DCC and DFR engaging in prosocial punishment in the 3:1 punishment ratio condition; n.s. *P* > 0.10, *χ*^2^ test. **c** The average expenditure of DCC and DFR on prosocial punishment in the 1:1 punishment ratio condition (*N*_DCC_ = 74; *N*_DFR_ = 41); n.s. *P* > 0.10, Mann–Whitney test. **d** Share of DCC and DFR engaging in prosocial punishment in the 1:1 punishment ratio condition; n.s. *P* > 0.10, *χ*^2^ test. The error bars indicate ± 1 SEM

We now examine each punishment condition separately (Fig. 3). When the punishment ratio was 3:1—which allows for the reduction of absolute payoff differences—the expenditures of DCC and DFR on prosocial punishment are nearly identical (*M*_DCC_ = 1.19, SD_DCC_ = 1.52; *M*_DFR_ = 1.19, SD_DFR_ = 1.60; Mann–Whitney *z* = −0.38, *P* = 0.707; Fig. 3a) and the difference in the proportion of prosocial punishers is not significant (60% DCC vs. 71% DFR; *χ*^2^(1) = 0.67, *P* = 0.412, *N* = 79; Fig. 3b).

The main point of interest in study 2 is the 1:1 punishment ratio condition, which excludes payoff-based motives for punishment, e.g. an aversion against or preference for inequality, because punishment does not change the inequality of payoffs. If the punishment of DFR is motivated primarily by inequality, they should not punish, or punish less than DCC, when the punishment ratio is 1:1. As in the 3:1 condition, DCC and DFR expenditures on prosocial punishment are very similar (*M*_DCC_ = 0.56, SD_DCC_ = 1.34; *M*_DFR_ = 0.68, SD_DFR_ = 1.35; Mann–Whitney *z* = −0.65, *P* = 0.513; Fig. 3c) and the proportion of prosocial punishers is indistinguishable (29% DCC vs. 39% DFR; *χ*^2^(1) = 0.84, *P* = 0.359, *N* = 79; Fig. 3d). Additionally, we do not find significant differences in the antisocial punishment behaviour of DCC and DFR (Supplementary Note 8). A series of regression models support these results and do not show evidence of differences in the frequency or severity of punishment between DCC and DFR, even when using a stricter classification of DCC, controlling for deviations from beliefs or using a continuous measure of strong positive reciprocity (Supplementary Note 9). Despite the stark difference in their disposition towards strong positive reciprocity, DCC and DFR seem to be strong negative reciprocators to the same degree, and, moreover, for a similar set of motives.

As discussed above, the 3:1 punishment ratio permits a larger set of motives for prosocial punishment. Accordingly, and in line with previous findings^56,57,59^, both DCC and DFR spent more on prosocial punishment when the punishment ratio was 3:1 than when it was 1:1 (Mann–Whitney *z* = 3.19, *P* = 0.001; *z* = 2.02, *P* = 0.043; resp.). Overall, independent of the disposition towards strong positive reciprocity, the 3:1 condition seems to tap into a larger range of motives to punish and induces higher punishment expenditure.

### Emotions (study 2)

**Fig. 4:**
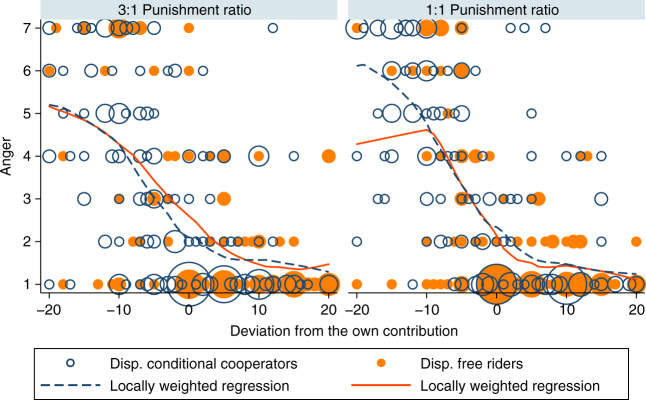
Self-reported anger increases for a negative deviation of others, independent of the punishment ratio or the individual cooperative disposition. The emotional reaction of DCC is similar to that of DFR in the 3:1 and 1:1 punishment ratio condition. The size of the bubbles corresponds to the number of observations at this location. The lines indicate the locally weighted regression functions for DCC (dark blue) and DFR (orange)

Similar to study 1, anger and punishment are positively correlated in both the 3:1 and 1:1 punishment ratio conditions (Supplementary Note 10). Figure 4 shows the anger levels reported by DCC and DFR separately for each punishment ratio. For both types and across the two ratios, anger is associated in a similar manner with negative deviations of other group members’ contributions. A regression analysis does not reveal significant level differences in anger across types for neither the 3:1 nor the 1:1 punishment ratios (Supplementary Note 10).

### Summary of study 2

Study 2 shows that independent of the punishment ratio and the set of relevant motives for punishment that it dictates, DCC and DFR use very similar levels of prosocial punishment, the type of punishment that drives and enables cooperation. Along with the replication of study 1’s finding that the relation between anger and punishment is similar for DCC and DFR, study 2 shows that DCC and DFR are not only very similar in their punishment behaviour, but are also guided by similar motives and emotional responses.

## Discussion

Our experiments, which measure cooperative dispositions independently from cooperation and punishment behaviour, provide unambiguous evidence for both strong positive and negative reciprocity, but do not support the hypothesis of a correlation between the two at the individual level. We find that the intrinsic disposition towards strong positive reciprocity (i.e. whether one is willing to positively reciprocate, even at a personal cost, others’ kind actions) is unrelated to the willingness to pay a cost in order to reciprocate others’ unkind actions by using punishment. In fact, in the presence of a punishment opportunity, DFR contribute only slightly less than DCC, and are very similar to them in their punishment levels, motives for punishment, and the way punishment is related to anger. Thus, selfishness does not extend to the realm of strong negative reciprocity; DFR do not free ride on the punishment of others. An important implication of our results is that the ‘burden of cooperation’ is carried by a larger set of individuals than previously assumed, which can help explain the high levels of cooperation observed when punishment opportunities are available^14,34,60–62^.

The distinction between people based on their disposition towards strong positive reciprocity, i.e. whether they are predisposed to conditionally cooperate with others or not, has proven to be crucial in understanding the dynamics of cooperation in the absence of punishment opportunities. In combination with expectations regarding the future contributions of others, measured dispositions help explain cooperation, which typically declines over time when the punishment of non-cooperators is not possible^5,33^. The current results suggest that the distinction between DCC and DFR is not crucial in explaining the maintenance of cooperation, which requires that there are sufficiently many strong negative reciprocators in the population. Since the punishment behaviour of DCC and DFR is virtually identical, the ongoing cooperative success of groups depends more on the presence of a sufficient number of strong negative reciprocators than on its composition in terms of DCC and DFR.

A likely explanation, which our data support, is that once an individual chooses to cooperate, be it due to a willingness to reciprocate the cooperation of others or due to fear of being punished for non-cooperation, the negative emotions associated with the free-riding behaviour of other group members, and the desire to relieve oneself from these emotions, take over strategic considerations^13^. In other words, some people are willing to exploit others, but nobody likes being exploited. This interpretation is consistent with Yamagishi et al.^41^ who find that rejections of unfair offers in ultimatum games are unrelated to prosocial cooperation in trust games and prisoner’s dilemma games. It is also consistent with experimental^34,40,42^ and survey-based evidence^38,39^ showing that behavioural measures consistent with strong positive and negative reciprocity are unrelated. Our experiments, which separate cooperative dispositions from behaviour, show that otherwise self-regarding people are therefore suddenly willing to bear the cost of disciplining wrongdoers, even in the absence of personal benefits for doing so.

A careful examination of the literature on social preferences reveals a number of frameworks that are suggestive of our results. For example, Fehr and Schmidt’s model of social preferences^23^ makes a distinction between attitudes towards disadvantageous and advantageous inequality, and models each with a separate parameter (*α* and *β*, respectively). An agent with *α* > 0 and *β* = 0 is expected to minimise disadvantageous inequality by punishing group members who contributed less than herself, but not to contribute to the public good even when expecting others to do so, because she does not mind the advantageous inequality. Such hypocritical punishment is exhibited by the DFR in our experiment who are willing to invest in punishing others. Note, however, that this reasoning holds only when punishment can indeed reduce disadvantageous inequality, as is the case in study 1 and when the punishment ratio was 3:1 in study 2. The willingness to punish when the punishment ratio is 1:1 is not readily explained by a desire to reduce inequality. Our results also question theories of social preferences that assume a single reciprocity parameter that governs behaviour in both the positive and negative reciprocity domain^20–22^.

Another example is recent work showing that there are two types of non-cooperators, namely Homo economicus and quasi-Homo economicus, who differ only slightly in the degree to which they pursue their own self-interest without regarding the welfare of others, or, in other words, in the degree to which they are non-reciprocal. These types, however, differ significantly in their psychological composition; while the former base their selfish choices on rational calculations, the latter are more impulsive^63^. This result shows that the non-reciprocal side of human behaviour is not unidimensional, but involves both choice patterns and psychological traits. Similarly, our data point to the conclusion that the reciprocal side of human behaviour is also not unidimensional; rather, there is a discontinuity when shifting from positive to negative reciprocity.

## Methods

### Participants and procedures (study 1)

We conducted the experiments between November 2012 and February 2014. We recruited 184 students at the University of Nottingham without prior experience in public goods experiments (101 females, average age = 19.84, SD = 2.14), using the recruitment software ORSEE^64^. The sample size was determined in expectation of heterogeneity in cooperative dispositions and to ensure a minimum number of participants from each type. From previous literature^4,65^, we expect about 25% of DFR. We therefore recruited at least 90 participants in each treatment to ensure a minimum expected number of 20 DFR. Participants were allocated to treatments at the session level; we conducted three sessions of the without punishment and six sessions of the with punishment treatment. The experiment was approved by the University of Nottingham School of Economics Ethics Committee and informed consent was obtained from all participants. The majority of participants were undergraduates from various fields of study (28% Humanities, 26% Economics and Business studies, 22% Natural Sciences and Engineering, 17% Law, Social and Political Sciences and 7% Medical Science). The experiment was computerised with z-Tree^66^. The experimental sessions lasted for about 90 minutes and participants’ earnings were paid in private at the end of each session (*M* = £10.25, SD = £2.00). Each session consisted of reading the instructions, computerised control questions, two experimental games and a questionnaire. The control questions were designed to check participants’ understanding of the games’ payoff functions. Participants had to correctly answer all control questions before the start of the experimental games. We did not provide any feedback after the first game in order to prevent participants from updating their beliefs as well as to exclude potential income effects and strategic play. See the Supplementary Note 11 for the instructions.

### The public goods game (study 1)

The core of our experimental design is a one-shot public goods game, played in groups of four. Each group member received an endowment of 20 tokens each, and decided how many tokens to keep for herself and how many to contribute to a common group project. Each token that a person kept for herself yielded one money unit (MU) to that person. Contributions to the project were multiplied by 1.6 and divided equally among the four group members. The social optimum is characterised by full contributions, whereas the individually money-maximising strategy is to contribute nothing, regardless of the choices of other group members.

The experiment included two phases, each with a different variation of the one-shot public goods game. The first phase was used to assess each participant’s individual disposition towards cooperation. In the second phase, groups were randomly re-matched and participants played a standard one-shot public goods game, either with or without punishment (in different treatments).

### First phase—measuring individual cooperative dispositions (study 1)

Individual dispositions towards cooperation were measured using the one-shot public goods game described above, played in a variant of the strategy method^4^. Each participant first decided on an unconditional contribution to the public good, and then on a series of conditional contributions, indicating her preferred contribution for each average (integer) contribution of the other group members. We refer to the resulting set of choices as the contribution schedule. For one randomly selected member of each group, the contribution schedule determined the actual contribution to the public good, ensuring that both decisions (unconditional and conditional contribution) are potentially payoff relevant. We also elicited participants’ beliefs about their group members’ average contribution: participants earned three MU for guessing the average contribution of others correctly, two MU for a deviation of one point, one MU for a deviation of two points, and zero MU for a higher deviation.

The contribution schedules were used to classify participants according to their disposition towards cooperation. The criteria were based on past work using this method^65^: DCC increase their contributions in the average contributions of others; they either have a positive Spearman’s rank correlation coefficient, significant at the 1% level, between their own contribution and the others’ average contribution, or display a monotonically increasing schedule, with at least one increase. DFR contribute exactly zero for each and every possible average contribution of others. Participants who were not classified as either DCC or DFR were excluded from the analysis, as our main hypothesis does not make unambiguous behavioural predictions for them.

### Second phase—cooperation and punishment behaviour (study 1)

In this phase, participants played the one-shot public goods game in direct-response mode either without or with punishment. In both treatments, each participant first decided, unconditional on the choices of others, how many tokens (out of 20) to contribute to the public good. In the without punishment treatment, beliefs about the group members’ average contribution were elicited and incentivised as in the first phase. Participants then learned the individual contribution of each of their group members in order to keep the information structure constant across the two treatments. Participants then proceeded to the emotion elicitation questionnaire described below. In the with punishment treatment, the belief elicitation was not incentivised to avoid punishment due to disappointment caused by lost income from inaccurate beliefs. After learning the individual contribution of each of their group members, participants had the option to assign up to five punishment points to each of their group members. Every punishment point cost the punisher one MU and destroyed two MU of the punished group member’s income. To avoid negative payoffs, each participant received a fixed payment of 10 MU. Then, participants stated their belief about how many punishment points they received from each of their group members and reported emotions as described below. Emotions were elicited before receiving feedback on the outcome of the punishment stage.

### Emotions elicitation (study 1)

Participants reported their emotional response to each group member’s individual contribution level. The questionnaire, adapted from Bosman and van Winden^51^, included thirteen emotions (anger, contempt, envy, fear, guilt, happiness, irritation, jealousy, joy, sadness, shame, surprise and warmth). Participants rated the intensity with which they felt each emotion on a seven-point scale ranging from 1 (not at all) to 7 (very much).

### Participants and procedures (study 2)

We conducted the experiments of study 2 between May and June 2016. The experimental procedures in study 2 were identical to those of study 1. Overall, 272 participants took part in study 2 (169 females, average age = 21.14, SD = 2.43). Like in study 1, we chose the sample size to account for heterogeneity of cooperative dispositions. However, we chose a significantly larger sample with 135 participants in the 3:1 and 137 participants in the 1:1 punishment ratio condition to increase the power to detect differences in punishment behaviour across dispositions. We conducted ten sessions in total and within each session participants were randomly allocated to either the 3:1 or the 1:1 punishment ratio condition. The majority of participants were undergraduates from various fields of study (12% Humanities, 13% Economics and Business studies, 29% Natural Sciences and Engineering, 15% Law, Social and Political Sciences and 31% Medical Science). Participants’ earnings were paid in private at the end of each session (*M* = £10.85, SD = £1.98).

### Random draw of the punishment ratio (study 2)

The first phase of study 2 was identical to that of study 1. The second phase closely follows the with punishment treatment in study 1. The difference was that in study 2 the punishment ratio was determined randomly. At the start of the game, participants were informed that each participant’s individual punishment ratio will be either 3:1 or 1:1, with a 50% chance for each option. A 3:1 (1:1) punishment ratio means that each punishment point cost a punishing participant one MU and destroyed three (one) MU of the punished group member’s income. The random draw was independent for each participant, such that individuals within each group could have different punishment ratios. This novel design allows for observing the effect of different punishment ratios on punishment while holding the contribution level constant.

### Statistical analysis

Our main results are based on standard non-parametric tests (Mann–Whitney tests, and Wilcoxon signed-rank tests) suitable to the nature of our experimental data. All tests are two-sided. Supplementary regression analyses are discussed in the Supplementary Information.

### Code availability

We used STATA/SE 15 for data analysis. The codes^67^ are available from the Dryad Digital Repository: 10.5061/dryad.dc42s75.

### Data availability

The data for the statistical analyses^67^ are available from the Dryad Digital Repository: 10.5061/dryad.dc42s75.

## Electronic supplementary material

Supplementary Information
