# Supplementary material for: Dispositional free riders do not free ride on punishment
Source: Nat Commun. 2018 Jun 19;9:2390. doi: 10.1038/s41467-018-04775-8 (PMC6008293; doi:10.1038/s41467-018-04775-8)
Supplement: Supplementary file 1 — Supplementary Information [file 41467_2018_4775_MOESM1_ESM.pdf]

Supplementary Information for

Dispositional free riders do not free ride on punishment

Weber *et al.*

This file includes:

Supplementary Note 1: Eliciting cooperative dispositions ………………………….……. p. 2

Supplementary Note 2: Consistency of cooperative dispositions (Study 1) ……………… p. 2

Supplementary Note 3: Antisocial punishment by DCC and DFR (Study 1) …………….. p. 4

Supplementary Note 4: Regression analysis of punishment (Study 1) …………………… p. 5

Supplementary Note 5: Analysis of emotions (Study 1) ………………………………… p. 10

Supplementary Note 6: Consistency of cooperative dispositions (Study 2) …………….. p. 14

Supplementary Note 7: Similarity of punishment by DCC and DFR …………………… p. 15

Supplementary Note 8: Antisocial punishment by DCC and DFR (Study 2) …………… p. 16

Supplementary Note 9: Regression analysis of punishment (Study 2) ………………….. p. 17

Supplementary Note 10: Analysis of emotions (Study 2) ……………………………….. p. 21

Supplementary Note 11: Experimental instructions ……………………………………... p. 24

Supplementary References ……………………………………………………………… p. 39

Supplementary Note 1: Eliciting cooperative dispositions

We use an anonymous one-shot public goods game, played with a variant of the strategy method, to measure cooperative dispositions, following the methodology introduced by Fischbacher et al.[1]. The details are described in the Methods section of the paper. The design allows to disentangle the disposition to cooperate from the belief about other group members' cooperative efforts, which is necessary to distinguish between types of players who would be indistinguishable from behavioural data alone. For example, in a one-shot public goods game played under strict anonymity, both a selfish player and a pessimistic strong positive reciprocator would contribute nothing.

The strategy method of classifying participants has several additional advantages. It closely resembles the tasks in the second phase of the experiment and was shown to successfully predict behaviour in similar games[2,3]. It has been replicated in different countries, showing that conditional cooperation and free riding are widespread cooperative dispositions across different societies[4-6]. Furthermore, there is evidence that the elicited contribution strategies are stable over time[7].

The method has been criticised[8], arguing that the variation in cooperative types may result from confusion regarding the game's payoff-maximising strategy rather than from underlying differences in preferences or dispositions to cooperate. However, conditional cooperation and free riding (DCC and DFR in the terminology we use) are also found by studies using other elicitation methods[9-11]. Additionally, a recent large-scale study (with over 2000 participants from the general population) found heterogeneous cooperative dispositions even after controlling for misperceptions of the payoff-maximising strategy[12].

Supplementary Note 2: Consistency of cooperative dispositions (Study 1)

Supplementary Fig. 1 shows the average contribution schedule for DCC and DFR as well as their individual contributions in the *Without Punishment* and *With Punishment* treatments. In both treatments, DCC contribute more if they hold higher beliefs, and their individual contributions are close to the average contribution schedule. The quadratic fitted regression lines are very close, indicating that contribution behaviour is similar in both treatments. Furthermore, contributions in both treatments are consistent with the predicted behaviour.

We calculate the predicted contribution using the individual contribution schedules from the first phase and beliefs from the second phase. 66% of DCC in the *Without Punishment* treatment and 59% of DCC in the *With Punishment* treatment are consistent with their contribution schedule (i.e., the deviation of the actual from the predicted contribution is at most ±2 MU). The consistency of DCC's contributions with their individual cooperative dispositions is similar across treatments (Kolmogorov-Smirnov equality-of-distributions test, $P = 0.400$). 81% of DFR in the *Without Punishment* treatment and 59% in the *With Punishment* treatment contribute exactly according to their indicated cooperative disposition. This difference in consistency is weakly significant (Kolmogorov-Smirnov equality-of-distributions test, $P = 0.099$), indicating that punishment has a larger effect on DFR than on DCC. DFR deviate more strongly from the contribution levels predicted by their individual cooperative dispositions. The quadratic fitted regression lines of Supplementary Fig. 1 illustrate that DFR increase their contribution with their beliefs about the group members' average contribution.

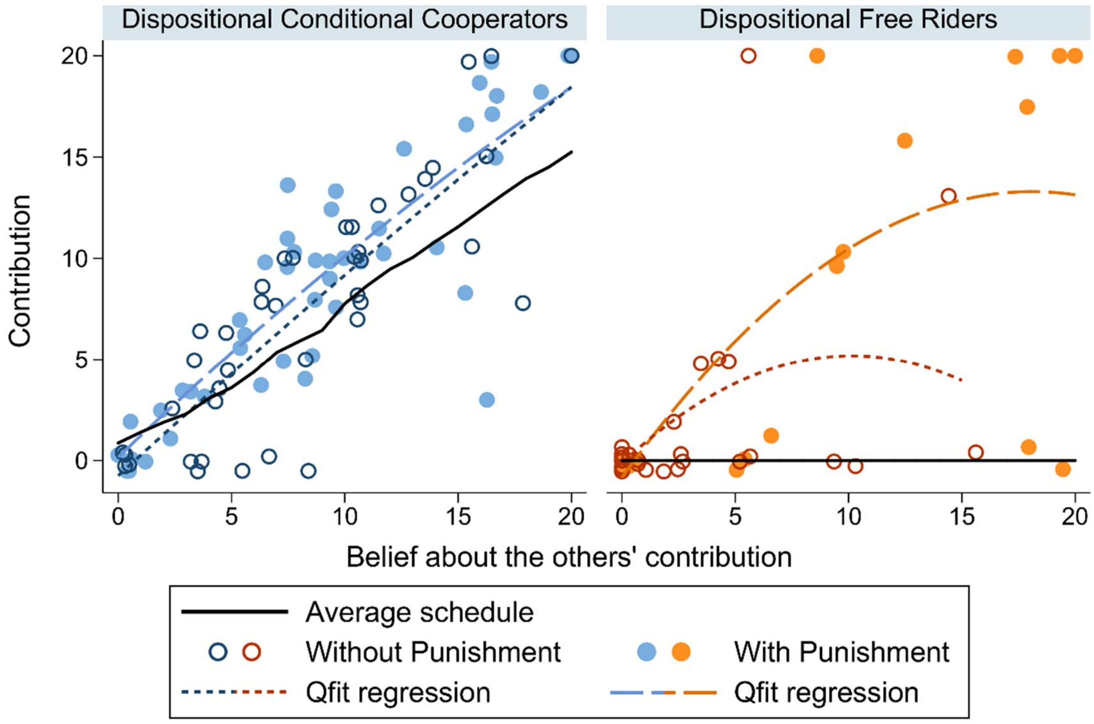

Supplementary Figure 1 | Consistency of behaviour with the disposition in Study 1. DCC (left panel) make higher contributions for a higher belief about other group members' average contribution. In both treatments, DCC act in line with their average schedule, elicited in the first part of the experiment. DFR (right panel) show a bigger deviation from their average schedule under the threat of punishment.

Supplementary Note 3: Antisocial punishment by DCC and DFR (Study 1)

Supplementary Fig. 2 shows a comparison of antisocial punishment in Study 1. Antisocial punishment refers to punishment that targets group members who contributed at least as much as the punisher[13]. Expenditure on antisocial punishment is slightly higher for DCC than DFR, but the difference is not significant ($M_{\mathrm{DCC}} = 0.04$, $SD_{\mathrm{DCC}} = 0.25$; $M_{\mathrm{DFR}} = 0.00$, $SD_{\mathrm{DFR}} = 0.00$; Mann-Whitney $z = 0.98$, $P = 0.326$; Supplementary Fig. 2a). We find similar proportions of antisocial punishers (5% DCC vs. 0% DFR; $\chi^2(1) = 0.98$, $P = 0.322$, $N = 56$; Supplementary Fig. 2b). A comparison of the above figures with those of prosocial punishment shows that engagement in antisocial punishment is very low in our sample.

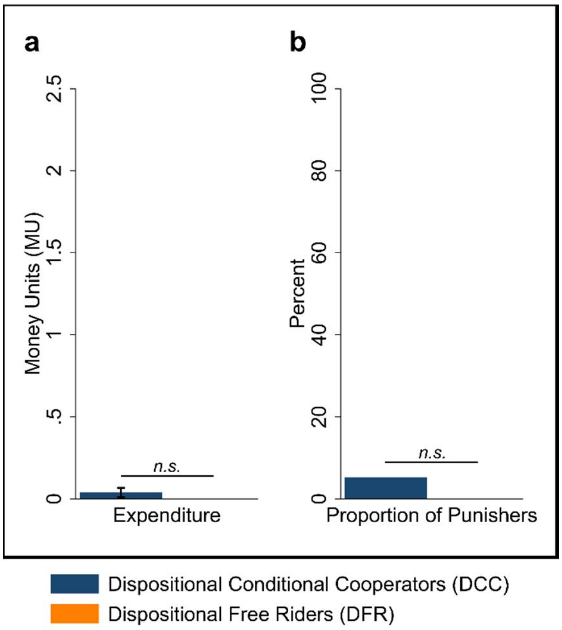

Supplementary Figure 2 | Antisocial punishment behaviour of Dispositional Conditional Cooperators (DCC) and Dispositional Free Riders (DFR). Mann-Whitney test for differences in the punishment expenditure with the subject as independent observation. χ^2 test for differences in the proportion of punishers. *n.s.* $P \geq 0.10$.

Supplementary Note 4: Regression analysis of punishment (Study 1)

Supplementary Table 1 reports the results of a regression analysis that controls for the magnitude of the deviation in subject *i*'s contribution with regard to group member *j*'s contribution and the behaviour of the other two group members. Both factors potentially influence the punishment decision. Inspired by previous research[14,*], we select a two-stage regression model to disentangle the subjects' likelihood to punish and the severity of punishment. However, the results are largely similar when running standard Tobit regressions. The first-stage regression (Col. 1) is a Probit model with a punishment dummy as dependent variable taking the value 0 if the subject does not engage in punishment and 1 otherwise. The second-stage regression includes only subjects who punish (Col. 3). We estimate a truncated linear regression with the number of punishment points as dependent variable, which accounts for the truncation at zero of the subsample. We include the same independent variables in the first and the second-stage regressions: The absolute negative deviation of group member *j*'s contribution from subject *i*'s (who is taking the punishment decision) contribution, the deviation of the other two group members' average contribution from subject *i*'s contribution, a dummy variable for DFR , as well as two interaction terms controlling for potential different reactions of DFR towards negative deviations in the contributions of the punished and the others'.

Col. 1 shows that the likelihood of punishment increases with a higher negative deviation of the targeted group member *j* from *i*'s contribution and for a higher average contribution deviation of other group members from *i*'s contribution. Neither the dummy variable DFR nor its interaction with the absolute negative contribution deviation of the two subjects are significant. This indicates that there is no level difference between the punishment chosen by DCC and DFR, and their reaction to a negative contribution deviation is similar. The interaction term 'DFR × The other group members' avg. contribution deviation from *i*' is negative and highly significant. Interestingly, the average marginal effect of this coefficient outweighs the positive coefficient for the others' contribution. We interpret this as opposing peer effects for DCC and DFR: DCC are more likely to punish a defector if the other group members contribute more on average to the public good. An explanation might be that a high average contribution of the others signals a high contribution norm to DCC, which they are

* We thank Nikos Nikiforakis for helpful discussions.

more likely to enforce. Conversely, DFR are less likely to punish a defector if the other group members contributed more on average.

The punishment severity (Col. 3) increases for higher negative deviations of the punished group member and for a higher average contribution of the other group members. Neither the dummy variable for DFR nor the interaction terms are statistically significant. This implies that once the decision to punish is made, there is no difference in the number of punishment points chosen by DCC and DFR.

The regression analyses reported in Col. 1-3 include, apart from DFR, subjects classified as DCC based on the criteria outlined in Fischbacher et al.[2]: The contribution schedule of DCC shows a positive and significant Spearman's rank correlation coefficient between the own conditional contribution and the contribution of others or a monotonically increasing schedule with at least one increase. This can be seen as a lenient definition of conditional cooperation, because it includes subjects whose contribution schedule deviates starkly from exactly matching the contributions of others ('perfect' conditional cooperation). Thus, we test whether the results reported above are sensitive to the definition of strong reciprocators by repeating the regression analysis for a more restrictive classification of DCC.

In Col. 4-6 of Supplementary Table 1, we exclude 'weak' DCC with a Spearman's rho ≤ 0.95 between the own conditional contribution and the contribution of others. Thus, the regression analysis includes only DFR and 'strong' DCC with a very large and highly significant Spearman's rank correlation coefficient, ensuring that these DCC are closer to perfect conditional cooperation. Applying this stronger definition of strong reciprocators leads to qualitatively similar results, with no level differences in the likelihood or severity of punishment between 'strong' DCC and DFR, demonstrating that our results are robust to a stricter definition of strong reciprocity.

Supplementary Table 1 | Two-stage regression model of punishment. The regression controls for the deviation in subject *i*'s contribution from group member *j*'s contribution.

	(1) Punishment decision	(2) Avg. marg. effects	(3) Punishment severity	(4) Punishment decision	(5) Avg. marg. effects	(6) Punishment severity
j's absolute negative contribution deviation from *i*	0.136*** (0.029)	0.028*** (0.006)	0.195*** (0.059)	0.152*** (0.034)	0.029*** (0.006)	0.179*** (0.056)
The other group members' average contribution deviation from *i*	0.052** (0.022)	0.011** (0.004)	0.069** (0.033)	0.059* (0.035)	0.011* (0.007)	0.014 (0.034)
Dispositional Free Rider (DFR)	−0.417 (0.457)	−0.085 (0.094)	−0.571 (0.790)	−0.401 (0.477)	−0.076 (0.090)	−0.271 (0.770)
DFR × *j*'s absolute negative contribution deviation from *i*	−0.045 (0.036)	−0.009 (0.007)	0.073 (0.094)	−0.061 (0.038)	−0.012* (0.007)	0.088 (0.100)
DFR × The other group members' avg. contribution deviation from *i*	−0.116*** (0.035)	−0.024*** (0.007)	0.032 (0.076)	−0.123*** (0.044)	−0.023*** (0.008)	0.087 (0.090)
Constant	−1.439*** (0.201)		0.970** (0.469)	−1.455*** (0.259)		0.705 (0.540)
N (Clusters)	213 (23)		43 (16)	147 (23)		31 (14)

Note. Col. 1, 4: Probit coefficients and punishment dummy as independent variable. Col. 2, 5: Average marginal effects of the Probit model. Col. 3, 6: Truncated linear regression with punishment points as independent variable. Col. 1-3: DCC and DFR included in the analysis. Col. 4-6: 'Weak' DCC with $r_s < 0.95$ excluded. *SE* clustered on groups are given in parentheses. * $P < 0.10$; ** $P < 0.05$; *** $P < 0.01$.

Additionally, we check for differences between DCC's and DFR's punishment behaviour when controlling for the deviations from *beliefs* (Supplementary Table 2). Thus, we re-estimate the two-stage regression model but now include the following independent variables: *j*'s absolute negative contribution deviation from *i*'s belief, the other group members average contribution deviation from *i*'s belief, a dummy variable for DFR and two interaction terms between DFR and the variables capturing the contribution deviations. The results are consistent with the findings on deviations from subject *i*'s contribution level. We find that the likelihood of punishment and the severity significantly increase with the deviation from beliefs. The likelihood and severity also increase with the deviation of group member *j* from the average contribution of the other two group members. However, the coefficient for DFR is not significantly different from zero, showing that there are no level differences in the likelihood and severity of punishment when controlling for the deviation from beliefs.

Supplementary Table 2 | Two-stage regression model of punishment. The regression controls for the deviation in subject *i*'s *belief* from group member *j*'s contribution.

Dependent variable: (1) punishment dummy; (3) punishment points	(1) Punishment decision	(2) Avg. marg. effects	(3) Punishment severity
j's absolute negative contribution deviation from *i*'s belief	0.147***<br>(0.030)	0.030***<br>(0.006)	0.157***<br>(0.051)
The other group members average contribution deviation from *i*'s belief	0.053**<br>(0.024)	0.011**<br>0.005	0.067**<br>(0.029)
Dispositional Free Rider (DFR)	−0.470<br>(0.407)	−0.094<br>0.081	−1.148<br>(0.992)
DFR × *j*'s absolute negative contribution deviation from *i*'s belief	−0.034<br>(0.046)	−0.007<br>0.009	0.144<br>(0.103)
DFR × The other group members average contribution deviation from *i*'s belief	−0.103**<br>(0.042)	−0.021**<br>0.008	0.039<br>(0.076)
Constant	−1.495***<br>(0.174)		1.268**<br>(0.530)
N (Clusters)	213 (23)		43 (16)

Note. Only DCC and DFR are included in the analysis. Col. 1: Probit coefficients; Col. 2: Average marginal effects of the Probit model; Col. 3: Truncated linear regression. *SE* clustered on groups are given in parentheses. * $P < 0.10$; ** $P < 0.05$; *** $P < 0.01$.

Is DCC's degree of positive reciprocity related to the likelihood of punishing? We use Spearman's rho between the own conditional contributions and the average contributions of others in the first phase as a measure of an individual's degree of positive reciprocity, which we refer to as the *Positive Reciprocity Score* (PRS). Larger positive values indicate a stronger tendency to reciprocate the contributions of others. Exploiting the variation in the PRS of DCC, we conduct an additional test of correlation between positive and negative reciprocity, which does not rely on the binary classification of types used above.

In particular, we conduct a two-stage regression analysis of punishment, similar to the one described above, but only including DCC (Supplementary Table 3). First, we explore the link between the PRS and the likelihood of engaging in punishment (Col. 1). The dependent variable is a punishment dummy and the independent variables include the absolute negative deviation of the punished group member, the average contribution deviation of the other group members and PRS. While the negative contribution deviation and the deviation from the contribution of others significantly predict the likelihood to engage in punishment, the PRS does not. This finding provides further evidence that strong positive and negative reciprocity are unrelated. We find a similar result for the punishment severity, which increases in the negative contribution deviation and the deviation from the average contribution of others but is not affected by PRS. This shows that even within the group of DCC, we do not find a significant link between the degree of strong reciprocity and the likelihood or severity of punishment.

Supplementary Table 3 | Correlation between positive and negative reciprocity.

Dependent variable: (1) punishment dummy; (3) punishment points	(1)<br>Punishment decision	(2)<br>Avg. marg. effects	(3)<br>Punishment severity
j's absolute negative deviation from *i*	0.138***<br>(0.030)	0.033***<br>(0.007)	0.205***<br>(0.062)
The other group members' average contribution deviation from *i*	0.050**<br>(0.023)	0.012**<br>(0.005)	0.070**<br>(0.034)
PRS	−0.742<br>(1.058)	−0.178<br>(0.257)	−0.936<br>(1.142)
Constant	−0.778<br>(0.991)		1.716<br>(1.167)
N (Clusters)	147 (22)		33 (14)

Note. Only DCC are included in the analysis. Col. 1: Probit coefficients; Col. 2: Average marginal effects of the Probit model; Col. 3: Truncated linear regression. *SE* clustered on groups are given in parentheses. * $P < 0.10$; ** $P < 0.05$; *** $P < 0.01$.

Supplementary Note 5: Analysis of emotions (Study 1)

Supplementary Table 4 shows that all five negative emotions included in the questionnaire are positively correlated with punishment expenditure, and their correlation coefficients are highly significant. Comparing the results for DCC and DFR separately reveals some differences: The number of punishment points that DFR choose is positively and significantly correlated with all five negative emotions. DCC exhibit a positive and significant correlation between punishment expenditure and negative emotions only for anger and irritation, but not for envy, jealousy and contempt. As envy and jealousy are payoff-oriented emotions, these results suggest that DFR do not only care about contribution norms but use punishment to reduce relative payoff differences.

Supplementary Table 4 | Spearman's rank correlation coefficient of negative emotions and punishment expenditure.

Emotion	Sample	r_s	*P*	*N*
Anger	All subjects	.45	< 0.001	276
	DCC	.48	< 0.001	147
	DFR	.38	0.002	66
Contempt	All subjects	.17	0.005	276
	DCC	.13	0.125	147
	DFR	.25	0.041	66
Envy	All subjects	.19	0.001	276
	DCC	.13	0.114	147
	DFR	.25	0.040	66
Irritation	All subjects	.37	< 0.001	276
	DCC	.33	< 0.001	147
	DFR	.34	0.005	66
Jealousy	All subjects	.16	0.010	276
	DCC	.10	0.218	147
	DFR	.34	0.006	66

Similar to Cubitt et al.[15], we define an 'emotions function' describing the self-reported intensity of a particular emotion by subject *i* with regard to a group member *j*. The intensity of the emotion depends on the difference in the contribution of *i* and *j*. For example, we expect a subject to report a higher anger level regarding one of her group members for a larger negative deviation of this group member's contribution compare to her own contribution.

Supplementary Table 5 provides the results of an ordered Probit regression model for the emotions functions. We test differences in the self-reported negative emotions between cooperative dispositions. The dependent variable (the intensity of the self-reported emotion reported by subject *i* with respect to group member *j*) is censored and restricted to integers from one to seven. The regression model controls for a positive and negative contribution deviation of group member *j* from subject *i*, as well as for the other two group members' behaviour. The model also includes a dummy variable for DFR. Furthermore, we include two interaction terms to allow for the possibility of differences in the slopes of the emotions functions of DCC and DFR. We estimate the regression separately for the *Without Punishment* treatment and the *With Punishment* treatment.

In the *Without Punishment* treatment, we find significant level differences between DCC and DFR for all negative emotions except for jealousy. Generally, DFR report a lower intensity of negative emotions compared to DFR when controlling for the negative deviation, positive deviation and the behaviour of other group members (except for jealousy).

In the *With Punishment* treatment, no significant level differences between DCC and DFR can be detected (except for jealousy). Looking at the regression model for anger, the only significant difference between types comes from DFR's reaction to positive deviations in the *With Punishment* treatment. DFR are significantly angrier than DCC if a group member contributed more than themselves. A reason might be the anticipation of punishment.

Supplementary Table 5 | Regression analysis of self-reported negative emotions.

	Without Punishment					*With Punishment*				
Dependent variable	Anger	Contempt	Envy	Irritation	Jealousy	Anger	Contempt	Envy	Irritation	Jealousy
j's absolute negative contribution deviation from *i*	0.080**<br>(0.035)	−0.016<br>(0.027)	0.054*<br>(0.029)	0.054*<br>(0.031)	0.048<br>(0.033)	0.168***<br>(0.043)	0.062**<br>(0.025)	0.058**<br>(0.029)	0.167***<br>(0.030)	0.061**<br>(0.029)
j's positive contribution deviation from *i*	−0.121**<br>(0.049)	−0.068**<br>(0.032)	−0.103**<br>(0.050)	−0.126***<br>(0.046)	−0.082*<br>(0.043)	−0.066**<br>(0.031)	0.025<br>(0.021)	−0.089***<br>(0.032)	−0.033<br>(0.024)	−0.096***<br>(0.031)
The other group members' average deviation from *i*	0.028*<br>(0.015)	0.001<br>(0.015)	0.015<br>(0.014)	0.020<br>(0.017)	−0.006<br>(0.013)	0.019<br>(0.015)	0.009<br>(0.010)	0.013<br>(0.015)	0.027**<br>(0.013)	0.019<br>(0.016)
Dispositional Free Rider (DFR)	−0.859*<br>(0.444)	−0.583*<br>(0.336)	−0.794**<br>(0.334)	−0.721*<br>(0.399)	−0.593<br>(0.386)	−0.111<br>(0.556)	−0.347<br>(0.386)	−0.788<br>(0.490)	−0.110<br>(0.421)	−0.885**<br>(0.441)
DFR × *j*'s absolute negative contribution deviation from *i*	0.022<br>(0.045)	0.073*<br>(0.040)	0.031<br>(0.038)	0.021<br>(0.046)	0.034<br>(0.048)	0.008<br>(0.063)	0.012<br>(0.052)	0.069<br>(0.043)	−0.012<br>(0.047)	0.058<br>(0.042)
DFR × *j*'s positive contribution deviation from *i*	0.105<br>(0.069)	0.097**<br>(0.043)	0.099<br>(0.061)	0.064<br>(0.068)	0.065<br>(0.060)	0.072**<br>(0.036)	−0.013<br>(0.022)	0.082<br>(0.059)	0.022<br>(0.037)	0.078<br>(0.056)
Pseudo R^2	0.11	0.02	0.08	0.10	0.09	0.21	0.03	0.11	0.17	0.11
N (Clusters)	204 (23)	204 (23)	204 (23)	204 (23)	204 (23)	213 (23)	213 (23)	213 (23)	213 (23)	213 (23)

Note. Only DCC and DFR included. Ordered Probit coefficients with robust *SE* clustered on groups. * $P < 0.10$; ** $P < 0.05$; *** $P < 0.01$.

Supplementary Note 6: Consistency of cooperative dispositions (Study 2)

Supplementary Fig. 3 shows the average contribution schedule from the first phase, as well as contributions and beliefs from the second phase of Study 2. The contributions of DCC are largely scattered around the diagonal and rising with beliefs. The quadratic fitted regression line is above the black line, illustrating the average cooperative dispositions of DCC. We find that 52% of DCC and 41% of DFR make a contribution in the second phase which is consistent with the predicted contribution using their contribution schedule from the first phase and their belief from the second phase (i.e., the deviation of the actual from the predicted contribution is at most ±2 MU).

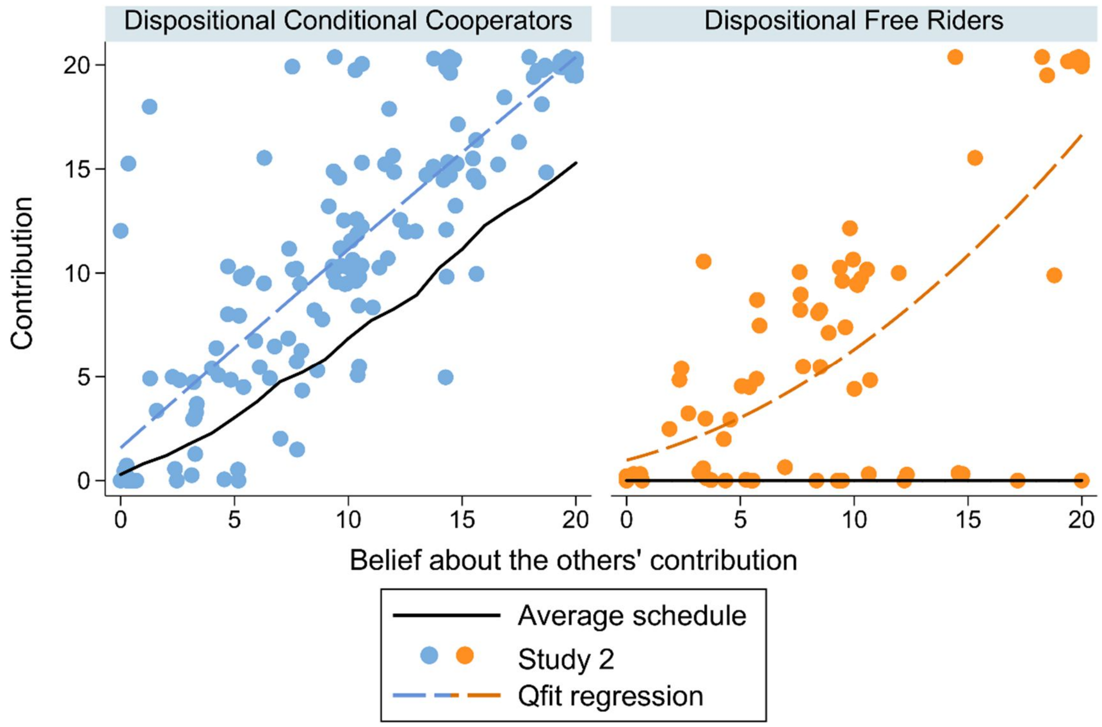

Supplementary Figure 3 | Consistency of behaviour with the disposition in Study 2. DCC (left panel) make higher contributions for higher beliefs about the other group members' average contribution. This is in line with their cooperative disposition indicated by the average schedule elicited in the first phase of Study 1. A large share of DFR (right panel) also increases the own contribution for a higher belief.

Supplementary Note 7: Similarity of punishment by DCC and DFR

We designed the punishment stage of Study 2 in a way that keeps the incentive to contribute constant across the two punishment ratios, 3:1 and 1:1. We chose the parameters so that the expected punishment ratio equals that of Study 1 (2:1). First, we compare the levels of punishment across Study 1 and 2. We find similar levels of prosocial punishment for both, DCC and DFR (Mann-Whitney $z_{DCC} = 1.41$, $P_{DCC} = 0.159$; $z_{DFR} = 0.43$, $P_{DFR} = 0.669$; subject as independent observation for all tests in this section). Additionally, the expenditure on antisocial punishment was comparable across Study 1 and 2 (Mann-Whitney $z_{DCC} = -0.72$, $P_{DCC} = 0.474$; $z_{DFR} = -1.40$, $P_{DFR} = 0.162$).

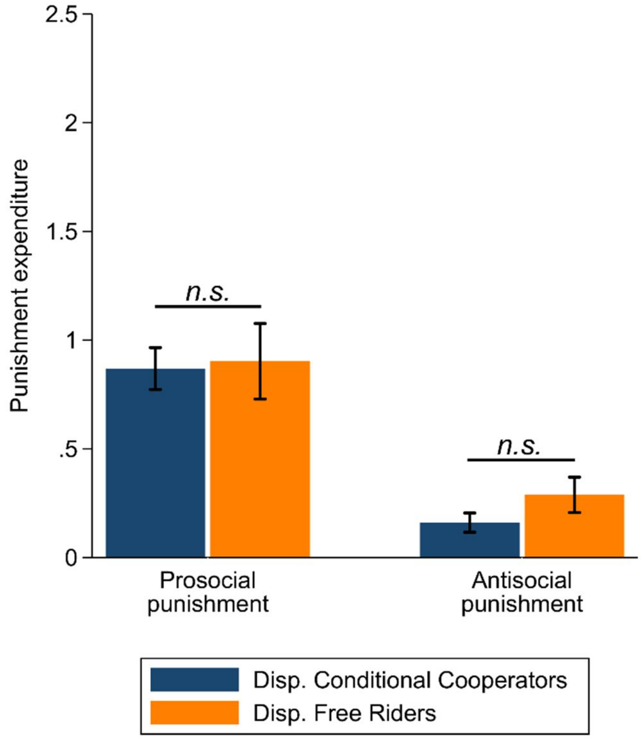

Supplementary Figure 4 | Average punishment in Study 2 is very similar for DCC and DFR. The 3:1 punishment ratio and the 1:1 punishment ratio conditions are pooled**.** This holds for both, prosocial and antisocial punishment. The error bars indicate bootstrapped ±1 *SEM*. Mann-Whitney test: *n.s.* $P \geq 0.10$; subject as independent observation.

We now pool the two conditions of Study 2 (Supplementary Fig. 4). We find similar punishment expenditures on prosocial punishment ($M_{DCC} = 0.87$, $SD_{DCC} = 1.46$; $M_{DFR} = 0.90$, $SD_{DFR} = 1.47$; Mann-Whitney $z = -0.34$, $P = 0.733$; subject as independent observation for all

tests in this section) and antisocial punishment DFR (M_{DCC} = 0.16, SD_{DCC} = 0.66; M_{DFR} = 0.29, SD_{DFR} = 1.02; Mann-Whitney $z = -0.39$, $P = 0.699$). We find similar frequencies of prosocial punishment (45% DCC vs. 53% DFR; $\chi^2(1) = 0.69$, $P = 0.406$, $N = 158$) and antisocial punishment (9% DCC vs. 10% DFR; $\chi^2(1) = 0.11$, $P = 0.742$, $N = 184$) comparing DCC and DFR.

Supplementary Note 8: Antisocial punishment by DCC and DFR (Study 2)

Supplementary Fig. 5 compares the antisocial punishment in Study 2 for the 3:1 and 1:1 punishment ratio condition separately. In the 3:1 punishment ratio condition, antisocial punishment is slightly higher for DFR than DCC, but the difference is not significant (M_{DCC} = 0.23, SD_{DCC} = 0.86; M_{DFR} = 0.41, SD_{DFR} = 1.08; Mann-Whitney $z = -1.24$, $P = 0.215$; Supplementary Fig. 5a). Additionally, differences in the proportions of antisocial punishers are not significant (10% DCC vs. 19% DFR; $\chi^2(1) = 1.56$, $P = 0.212$, $N = 94$; Supplementary Fig. 5b).

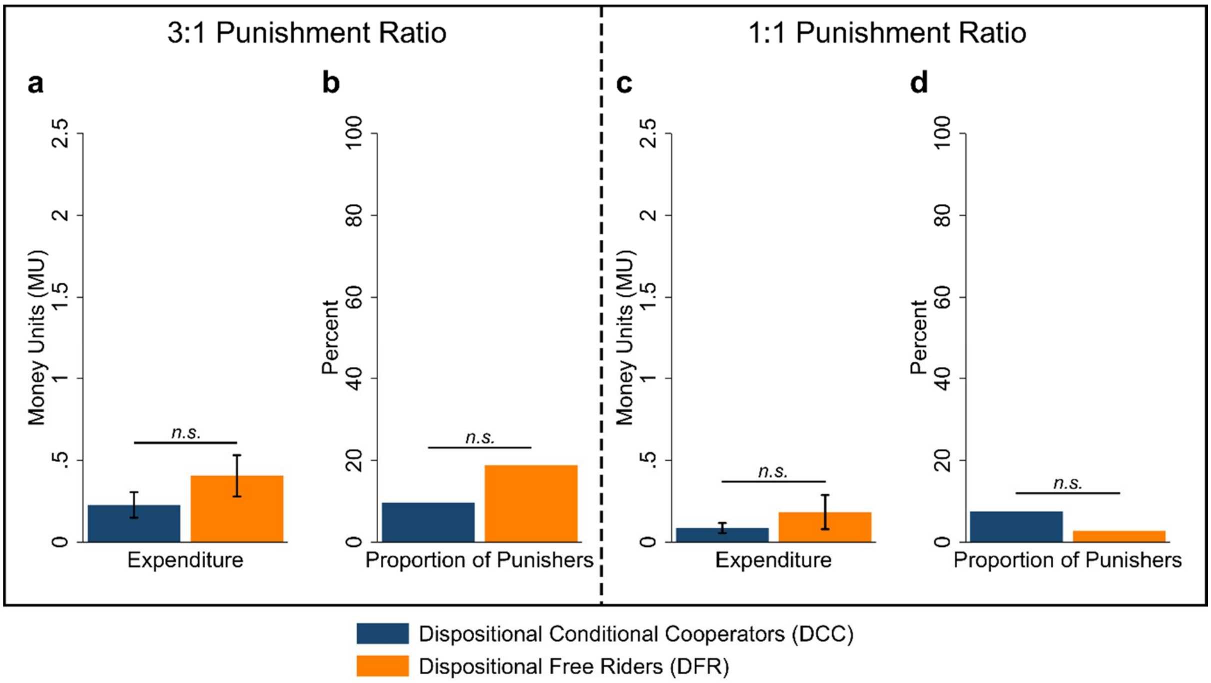

Supplementary Figure 5 | Antisocial punishment behaviour of Dispositional Conditional Cooperators (DCC) and Dispositional Free Riders (DFR) in the 3:1 Punishment Ratio condition and the 1:1 punishment ratio condition. Mann-Whitney test for differences in the punishment expenditure with the subject as independent observation. χ^2 test for differences in the proportion of punishers. *n.s.* $P \geq 0.10$.

Similarly, in the 1:1 punishment ratio condition the expenditures on antisocial punishment of DCC and DFR do not significantly differ ($M_{\text{DCC}} = 0.09$, $SD_{\text{DCC}} = 0.31$; $M_{\text{SR}} = 0.18$, $SD_{\text{DFR}} = 0.94$; Mann-Whitney $z = 0.94$, $P = 0.347$; Supplementary Fig. 5c). The proportion of antisocial punishers is similar for DCC and DFR (8% DCC vs. 3% DFR; $\chi^2(1) = 0.97$, $P = 0.324$, $N = 90$; Supplementary Fig. 5d).

Supplementary Note 9: Regression analysis of punishment (Study 2)

We use the same two-stage regression model as in Study 1, reported in Supplementary Table 1, to disentangle potential differences in the likelihood and severity of punishment between DCC and DFR. In both, the 3:1 and 1:1 punishment ratio condition, the likelihood and severity of punishment are positively associated with a larger negative deviation from subject *i*'s contribution (Supplementary Table 6). The same holds true for a larger deviation between subject *i*'s contribution and the other two group members' average contribution deviation. The only exception is the punishment severity in the 1:1 condition for which the other group members' average contribution deviation from *i*'s contribution has no significant effect. The insignificant dummy variable for DFR shows that there are no level differences in the likelihood or severity of punishment between DCC and DFR. This is true for both the 3:1 and the 1:1 conditions. In 1:1, the severity of punishment inflicted by DFR is significantly higher for a larger deviation of the other two group members, as compared to DCC.

We repeated the above analysis applying a stricter definition of strong reciprocity (Supplementary Table 7). The results are qualitatively similar to those obtained with our initial definition of strong reciprocity, showing that they are robust to a stricter definition of strong reciprocity.

Supplementary Table 6 | Two-stage regression model of punishment.

	3:1			*1:1*		
	(1) Punishment decision	(2) Avg. marg. effects	(3) Punishment severity	(4) Punishment decision	(5) Avg. marg. effects	(6) Punishment severity
j's absolute negative contribution deviation from *i*	0.119*** (0.023)	0.035*** (0.006)	0.203*** (0.076)	0.090*** (0.024)	0.017*** (0.004)	0.134** (0.066)
The other group members' average contribution deviation from *i*	0.029* (0.017)	0.008* (0.005)	0.123*** (0.031)	0.043** (0.017)	0.008** (0.003)	−0.049 (0.073)
Dispositional Free Rider (DFR)	−0.061 (0.350)	−0.018 (0.102)	1.309 (2.152)	−0.455 (0.371)	−0.084 (0.074)	0.518 (1.382)
DFR × *j*'s absolute negative contribution deviation from *i*	−0.007 (0.053)	−0.002 (0.016)	−0.127 (0.200)	0.080* (0.048)	0.015 (0.009)	0.082 (0.143)
DFR × The other group members' average contribution deviation from *i*	0.022 (0.028)	0.006 (0.008)	−0.186 (0.137)	0.022 (0.039)	0.004 (0.007)	0.229** (0.116)
Constant	−1.065*** (0.162)		0.325 (0.759)	−1.574*** (0.265)		0.556 (0.676)
N (Clusters)	336 (63)		101 (45)	345 (61)		46 (24)

Note. DCC and DFR included in the analysis. Col. 1, 4: Probit coefficients and punishment dummy as independent variable. Col. 2, 5: Average marginal effects of the Probit model. Col. 3, 6: Truncated linear regression with punishment points as independent variable. *SE* clustered on groups are given in parentheses. * $P < 0.10$; ** $P < 0.05$; *** $P < 0.01$.

Supplementary Table 7 | Two-stage regression model of punishment (‘weak’ DCC excluded).

	3:1			*1:1*		
	(1) Punishment decision	(2) Avg. marg. effects	(3) Punishment severity	(4) Punishment decision	(5) Avg. marg. effects	(6) Punishment severity
j’s absolute negative contribution deviation from *i*	0.134*** (0.028)	0.039*** (0.007)	0.231*** (0.069)	0.112*** (0.036)	0.017*** (0.005)	0.082 (0.082)
The other group members’ average contribution deviation from *i*	0.056*** (0.021)	0.016*** (0.006)	0.139*** (0.032)	0.021 (0.033)	0.003 (0.005)	−0.114** (0.044)
Dispositional Free Rider (DFR)	−0.012 (0.362)	−0.003 (0.104)	1.915 (1.879)	−0.079 (0.454)	−0.012 (0.069)	−0.266 (1.491)
DFR × *j*’s absolute negative contribution deviation from *i*	−0.023 (0.055)	−0.007 (0.016)	−0.161 (0.180)	0.058 (0.055)	0.009 (0.009)	0.124 (0.149)
DFR × The other group members’ average contribution deviation from *i*	−0.006 (0.030)	−0.002 (0.009)	−0.196 (0.124)	0.045 (0.049)	0.007 (0.008)	0.285*** (0.096)
Constant	−1.113*** (0.194)		−0.095 (0.625)	−1.950*** (0.367)		1.492* (0.856)
N (Clusters)	267 (58)		81 (40)	243 (51)		27 (17)

Note. ‘Weak’ DCC with $r_s \leq 0.95$ excluded. Col. 1, 4: Probit coefficients and punishment dummy as independent variable. Col. 2, 5: Average marginal effects of the Probit model. Col. 3, 6: Truncated linear regression with punishment points as independent variable. *SE* clustered on groups are given in parentheses. * $P < 0.10$; ** $P < 0.05$; *** $P < 0.01$.

We find similar results when basing our regression model on the deviation of group member *j*'s contribution from subject *i*'s *beliefs*, rather than her actual contributions (Supplementary Table 8). We do not find significant level differences between DCC and DFR for the likelihood or severity of punishment in neither the 3:1 nor 1:1 conditions.

Supplementary Table 8 | Two-stage regression model of punishment depending on deviation from the punisher's *belief* about other group members' average contributions.

	3:1		*1:1*	
	(1) Punishment decision	(2) Punishment severity	(3) Punishment decision	(4) Punishment severity
j's absolute neg. contribution deviation from *i*'s belief	0.134*** (0.025)	0.185** (0.078)	0.103*** (0.025)	0.112 (0.089)
The others' average contribution deviation from *i*	0.024 (0.019)	0.116*** (0.032)	0.046** (0.019)	−0.015 (0.099)
Dispositional Free Rider (DFR)	−0.078 (0.371)	0.156 (2.223)	−0.130 (0.417)	2.271 (1.790)
DFR × *j*'s absolute neg. deviation from *i*'s belief	−0.012 (0.050)	−0.031 (0.209)	0.024 (0.054)	−0.157 (0.204)
DFR × The others' average contribution deviation from *i*	0.045 (0.032)	−0.067 (0.120)	−0.030 (0.031)	−0.049 (0.135)
Constant	−1.073*** (0.164)	0.464 (0.750)	−1.607*** (0.260)	0.768 (0.860)
N (Clusters)	336 (63)	101 (45)	345 (61)	46 (24)

Note. Includes only DCC and DFR. Col. 1, 3: Probit coefficients with punishment dummy as dependent variable; Col. 2, 4: Truncated linear regression with punishment points as dependent variable. Robust *SE* clustered on groups are given in parentheses. * $P < 0.10$; ** $P < 0.05$; *** $P < 0.01$.

Similar to Study 1, we test for a link between the degree of positive reciprocity (PRS) which DCC display and their punishment decisions (Supplementary Table 9). Surprisingly, for the 3:1 punishment ratio condition, the degree of positive reciprocity is negatively (and significantly) related to the likelihood and severity of punishment. This suggests that subjects closer to perfect conditional cooperation are less likely to punish than others, and when they

do, their punishment is less severe. We do not find any significant association between the degree of positive reciprocity and punishment in the 1:1 punishment ratio condition.

Supplementary Table 9 | Correlation between positive and negative reciprocity.

	3:1		*1:1*	
	(1) Punishment decision	(2) Punishment severity	(3) Punishment decision	(4) Punishment severity
j's absolute neg. contribution deviation from *i*'s belief	0.123*** (0.023)	0.215*** (0.051)	0.090*** (0.024)	0.136** (0.069)
The others' average contribution deviation from *i*	0.028 (0.017)	0.132*** (0.022)	0.043** (0.018)	−0.046 (0.070)
PRS	−2.415** (1.043)	−4.826* (2.737)	0.166 (1.058)	2.882 (4.204)
Constant	1.179 (0.988)	4.844* (2.828)	−1.726* (1.046)	−2.163 (4.002)
N (Clusters)	231 (53)	71 (31)	222 (48)	32 (16)

Note. Only DCC are included in the analysis. Col. 1, 3: Probit coefficients; Col. 2, 4: Truncated linear regression. *SE* clustered on groups are given in parentheses. * $P < 0.10$; ** $P < 0.05$; *** $P < 0.01$.

Supplementary Note 10: Analysis of emotions (Study 2)

Supplementary Table 10 explores the link between negative emotions and punishment expenditure. It reports the Spearman's rank correlation coefficient for all five negative emotions and punishment expenditure. Pooling all subjects shows that negative emotions are generally positively and significantly associated with punishment expenditure. Similar to Study 1, we find that anger and irritation have the largest correlation coefficients, and they are highly significant.

Supplementary Table 10 | Spearman's rank correlation coefficient of negative emotions and punishment expenditure in Study 2.

		3:1			*1:1*		
Emotion	Sample	r_s	*P*	*N*	r_s	*P*	*N*
	All subjects	.46	< 0.001	405	.34	< 0.001	411
Anger	DCC	.47	< 0.001	231	.22	< 0.001	222
	DFR	.40	< 0.001	105	.53	< 0.001	123
	All subjects	.19	< 0.001	405	.21	< 0.001	411
Contempt	DCC	.23	< 0.001	231	.07	0.269	222
	DFR	.06	0.535	105	.30	< 0.001	123
	All subjects	.19	< 0.001	405	.26	< 0.001	411
Envy	DCC	.18	0.007	231	.17	0.010	222
	DFR	.11	0.273	105	.40	< 0.001	123
	All subjects	.43	< 0.001	405	.29	< 0.001	411
Irritation	DCC	.46	< 0.001	231	.21	0.002	222
	DFR	.29	0.003	105	.46	< 0.001	123
	All subjects	.20	< 0.001	405	.27	< 0.001	411
Jealousy	DCC	.21	0.001	231	.13	0.051	222
	DFR	.01	0.933	105	.45	< 0.001	123

Supplementary Table 11 reports the result of a regression analysis to investigate the emotions functions in the 3:1 and the 1:1 conditions. Like in Study 1 this helps to investigate the subjects' negative emotions as a driving factors behind punishment. Controlling for absolute positive and negative contribution deviations, as well as the average contribution deviation of the other group members, we generally find no level differences in the intensity of self-reported negative emotions comparing DCC and DFR. A notable exception is that DFR report lower jealousy, compared to DCC, in the 1:1 condition, and this difference is weakly significant.

Supplementary Table 11 | Regression analysis of self-reported negative emotions.

	3:1					*1:1*				
Dependent variable	Anger	Contempt	Envy	Irritation	Jealousy	Anger	Contempt	Envy	Irritation	Jealousy
j's absolute negative contribution deviation from *i*	0.126***<br>(0.018)	0.039**<br>(0.019)	0.072***<br>(0.019)	0.134***<br>(0.019)	0.060***<br>(0.018)	0.173***<br>(0.020)	0.049***<br>(0.018)	0.073***<br>(0.021)	0.153***<br>(0.020)	0.074***<br>(0.021)
j's positive contribution deviation from *i*	−0.051*<br>(0.028)	−0.016<br>(0.022)	−0.019<br>(0.022)	−0.055**<br>(0.027)	−0.008<br>(0.024)	−0.051*<br>(0.027)	−0.005<br>(0.022)	−0.059**<br>(0.028)	−0.060**<br>(0.028)	−0.049**<br>(0.023)
The other group members' average deviation from *i*	0.019*<br>(0.012)	−0.007<br>(0.011)	−0.008<br>(0.011)	0.020*<br>(0.011)	−0.018<br>(0.012)	0.020*<br>(0.010)	0.016<br>(0.011)	0.020<br>(0.013)	0.014<br>(0.011)	0.022*<br>(0.013)
Dispositional Free Rider (DFR)	0.207<br>(0.307)	0.038<br>(0.256)	0.165<br>(0.299)	−0.099<br>(0.331)	0.045<br>(0.323)	−0.106<br>(0.287)	−0.096<br>(0.312)	−0.230<br>(0.285)	0.129<br>(0.284)	−0.576*<br>(0.346)
DFR × *j*'s absolute negative contribution deviation from *i*	−0.010<br>(0.049)	−0.039<br>(0.046)	−0.007<br>(0.038)	0.044<br>(0.046)	−0.004<br>(0.040)	−0.001<br>(0.066)	0.003<br>(0.050)	0.072<br>(0.058)	−0.002<br>(0.065)	0.095<br>(0.061)
DFR × *j*'s positive contribution deviation from *i*	−0.010<br>(0.042)	0.012<br>(0.030)	−0.015<br>(0.039)	0.015<br>(0.037)	0.003<br>(0.040)	0.021<br>(0.034)	0.041<br>(0.030)	0.055<br>(0.036)	0.016<br>(0.037)	0.076**<br>(0.033)
Pseudo R^2	0.14	0.02	0.07	0.17	0.05	0.21	0.02	0.08	0.19	0.08
N (Clusters)	336 (63)	336 (63)	336 (63)	336 (63)	336 (63)	345 (61)	345 (61)	345 (61)	345 (61)	345 (61)

Note. Only DCC and DFR included. Ordered Probit coefficients with robust *SE* clustered on groups. * $P < 0.10$; ** $P < 0.05$; *** $P < 0.01$.

Supplementary Note 11: Experimental instructions

Study 1: Without Punishment treatment – Part 1

You are now taking part in an economic experiment. Depending on the decisions made by you and other participants, you can earn a considerable amount of money. It is therefore very important that you read these instructions with care.

These instructions are solely for your private use. **It is prohibited to communicate with other participants during the experiment.** If you have any questions, please raise your hand. A member of the experiment team will come and answer them in private. If you violate this rule, you will be dismissed from the experiment and you will forfeit all payments.

During the experiment, we will not speak in terms of Pounds, but in Guilders. At the end your entire earnings will be calculated in Guilders. The total amount of Guilders you have earned will be converted to Pounds at the following rate:

1 Guilder = 0.20 Pounds

After this experimental session, your entire earnings from the experiment will be paid to you privately in cash.

At the end of the session, you will be asked to fill in a questionnaire. The answers you provide in this questionnaire are completely anonymous. They will not be revealed to anyone either during the experiment or after it. Furthermore, your responses to the questionnaires will not affect your earnings during the experiment.

The groups

At the beginning of the experiment, all participants will be randomly divided into groups of four. Apart from you, there will be three other members in your group. **You will not learn who the other people in your group are at any point.**

The decision situation

Each participant receives an endowment of **20 tokens**. You have to decide how many of these 20 tokens you will contribute to a group project, and how many you will keep for yourself. The three other members of your group have to make the same decision. They can also either contribute tokens to the project or keep tokens for themselves. You and the other members of

the group can each choose any amount between 0 and 20 tokens to contribute (including 0 and 20).

The payoffs

The income of every member of the group is calculated in the same way. Your income consists of two components:

(1) The first component is the amount of tokens that you keep for yourself. Every token that you do not contribute to the project automatically belongs to you and earns you one Guilder.

(2) The second component is your personal return from the group project. For all of the tokens contributed to the project the following happens: the project's value will be multiplied by 1.6 and this amount will be divided equally among all four members of the group.

For example, if 1 token is contributed to the project, the project's value increases to 1.6 Guilders. This amount is divided equally among all four members of the group. Thus every group member receives 0.4 Guilders.

The following function illustrates your income in Guilders:

$$\textit{Your Total Income} = 20 - \textit{Your Contribution} + 0.4 \times (\textit{Group Project})$$

In order to explain the income calculation we will give some examples. Please read them carefully. At the end of the introductory information, you will be asked to answer several computerised control questions which are designed to check that you have understood the decision situation.

Example 1

If each of the four members of the group contributes 0 tokens to the project, all four will receive an income from their private account of 20. Nobody receives anything from the project, because no one contributed anything. Therefore the total income of every member of the group is 20 Guilders.

Calculation of the total income of every participant: $(20 - 0) + 0.4 \times (0) = 20$

Example 2

If each of the four members of the group contributes 20 tokens, there will be a total of 80 tokens contributed to the project. The income from the private account is 0 for everyone, but each member receives an income from the project of $0.4 \times 80 = 32$ Guilders.

Calculation of the total income of every participant: $(20 - 20) + 0.4 \times (80) = 32$

Example 3

If you contribute 20 tokens, the second member 10 tokens, the third member 5 and the fourth 0 tokens, the following incomes are calculated:

Because the total contribution to the project is 35 tokens, everyone will receive $0.4 \times 35 = 14$ Guilders from the project.

You contributed all your 20 tokens to the project. You will therefore receive 14 Guilders in total at the end of the experiment.

The second member of the group also receives 14 Guilders from the project. In addition, she receives 10 Guilders from her private account, because she contributed 10 tokens to the project. Thus, her total income is 24 Guilders altogether.

The third group member receives 14 Guilders from the project as well. Additionally, this group member will receive 15 Guilders from her private account. The total income therefore adds up to 29 Guilders.

The fourth member of the group, who did not contribute anything, also receives the 14 Guilders from the project and additionally the 20 Guilders from the private account, which means her total income is 34 Guilders.

Calculation of your total income: $(20 - 20) + 0.4 \times (35) = 14$
Calculation of the 2nd group member's total income: $(20 - 10) + 0.4 \times (35) = 24$
Calculation of the 3rd group member's total income: $(20 - 5) + 0.4 \times (35) = 29$
Calculation of the 4th group member's total income: $(20 - 0) + 0.4 \times (35) = 34$

Example 4

The three other members of your group contribute 20 tokens each to the project. You do not contribute anything. In this case the incomes will be calculated as follows:

Calculation of your total income: $(20 - 0) + 0.4 \times (60) = 44$
Calculation of the total income of each other group member: $(20 - 20) + 0.4 \times (60) = 24$

The experiment

The experiment is based on the decision situation just described to you, conducted **only once**. In this experiment you will make two types of decisions: an **unconditional contribution** and filling in a **contribution table**.

When making your **unconditional contribution**, the following screen will appear:

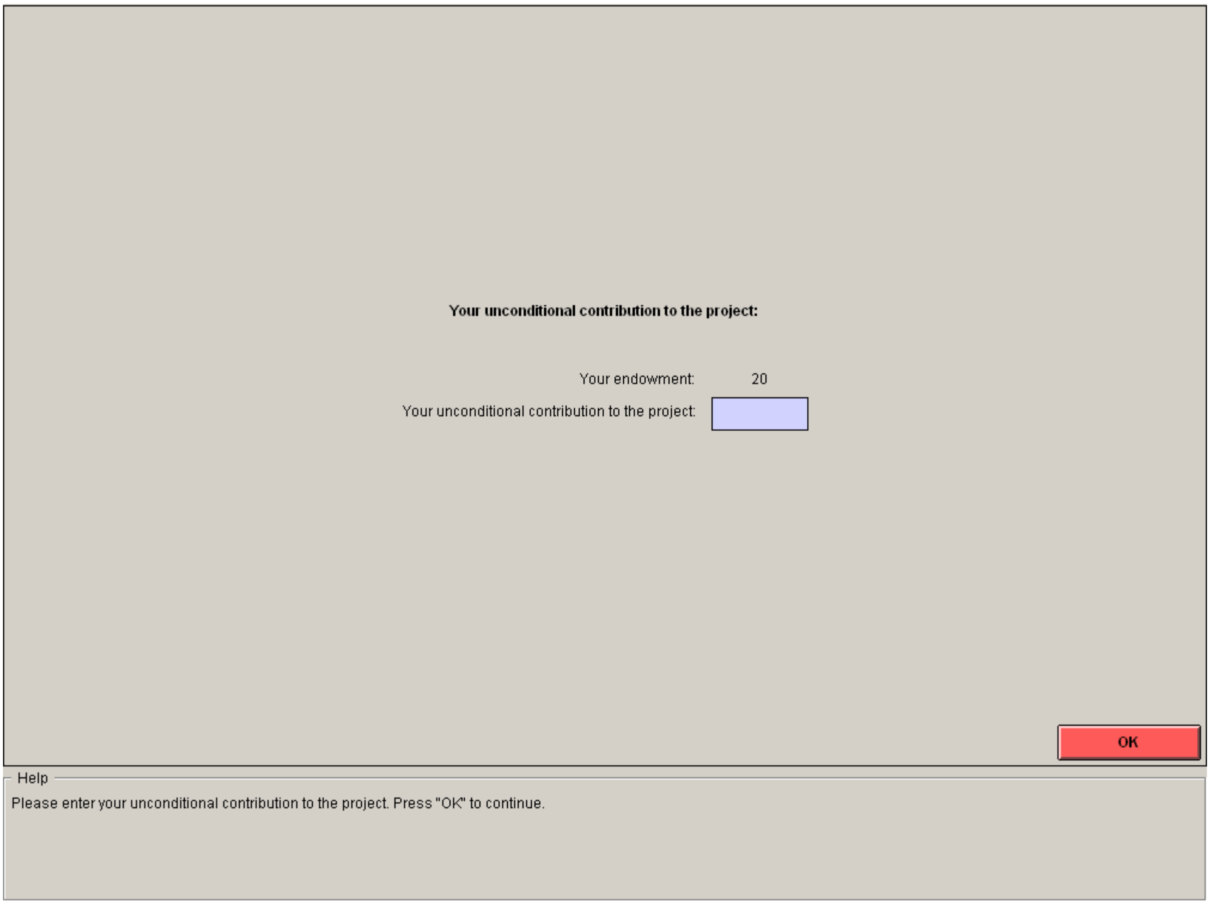

As mentioned above, your endowment in the experiment is 20 tokens. You have to decide how many tokens you contribute to the project by typing a number between 0 and 20 (including 0 and 20) in the box. This box can be reached by clicking on it with the mouse. By deciding how

many tokens to contribute to the project, you automatically decide how many tokens you keep for yourself. After entering the amount of tokens you want to contribute you must click on the "*OK*" button. Once you have done this, your decision can no longer be revised.

Your second task is to fill in a **contribution table** on the following screen:

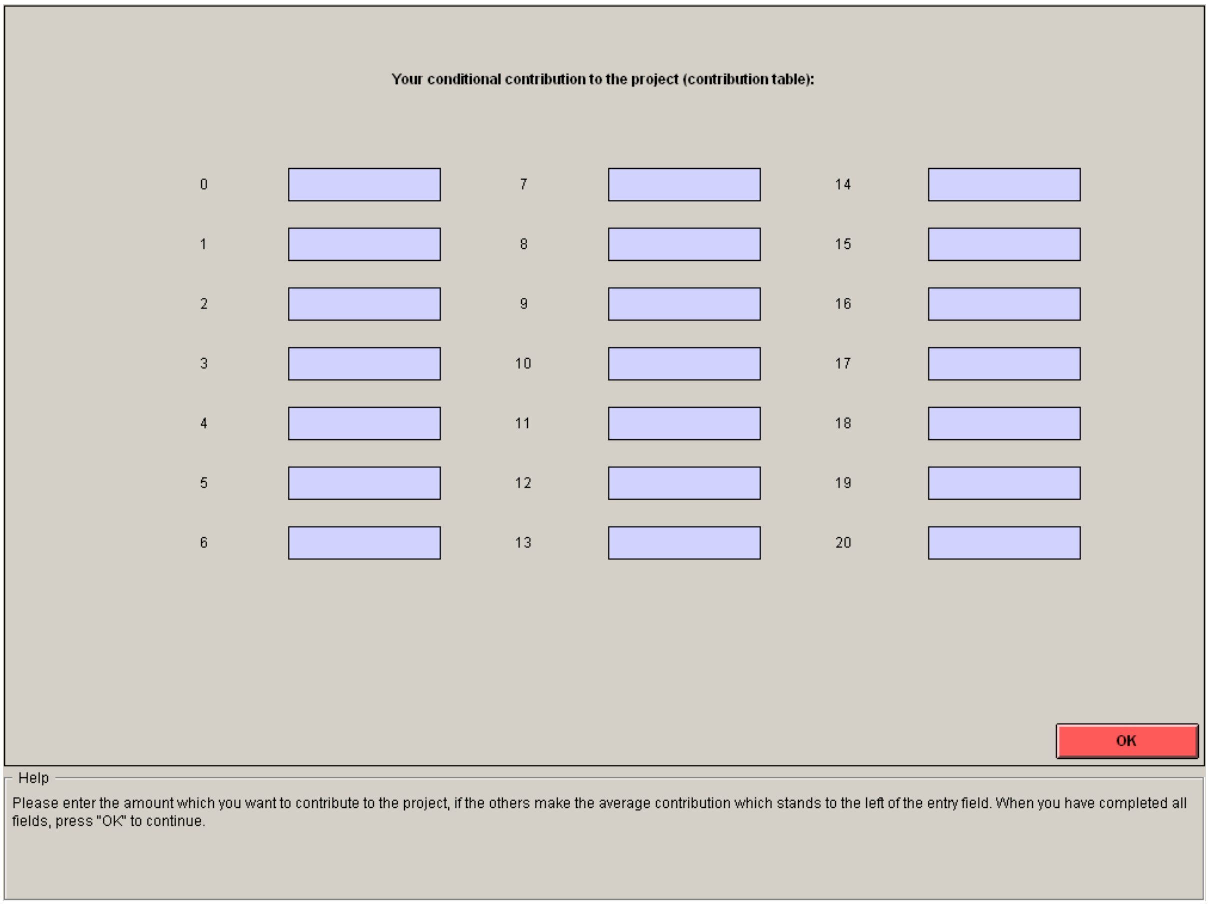

The contribution table indicates **how many tokens you want to contribute to the project for each possible average contribution of the other group members** (rounded to the nearest integer). The table allows for conditioning your contribution on that of the other group members.

The numbers to the left of the input fields are the possible average contributions of the **other** group members (rounded to the nearest integer). You have to enter how many tokens you want to contribute to the project, conditional on the indicated average contribution of the other group members. **You must enter a number between 0 and 20 (including 0 and 20) into each box.**

For example, in the first box you enter the amount of tokens you want to contribute to the project in case the average contribution to the project of the other three group members is 0

tokens. In the next boxes you enter how much you contribute for an average contribution of 1, 2, 3, … tokens. After entering your decisions, you must click on the "*OK*" button.

After all participants of the experiment have made an unconditional contribution and have filled their contribution table, a random mechanism will select one member from every group. For **this group member, the contribution table** will be used to determine the contribution to the project. Whereas for **the other three group members, their unconditional contributions** will define the amount of tokens they add to the project.

You will not know whom the random mechanism will select before you make your unconditional contribution and fill in the contribution table. Therefore you must think carefully about both decisions. Either of them could determine your actual contribution to the project.

Example 5

Suppose that the **random mechanism selects you**; and that the other three group members made unconditional contributions of 0, 2, and 4 tokens, respectively. The average contribution of these three group members is, therefore, 2 tokens. If you indicated in your contribution table that you will contribute 1 token if the others contribute 2 tokens on average, then the total contribution to the project is given by 0 + 2 + 4 + 1 = 7 tokens. Each group member would, therefore, earn 0.4 × 7 = 2.8 Guilders from the project plus their respective income from their own private account. If, instead, you indicated in your contribution table that you would contribute 19 tokens if the others contribute 2 tokens on average, then the total contribution of the group to the project would be given by 0 + 2 + 4 + 19 = 25 tokens. Each group member would earn 0.4 × 25 = 10 Guilders from the project plus their respective income from their own private account.

Example 6

Suppose that the **random mechanism does not select you**; and that your unconditional contribution is 16 tokens, while those of the other two group members not selected by the random mechanism are 18 and 20 tokens respectively. Your average unconditional contribution and that of these two other group members is, therefore, 18 tokens. If the group member whom the random mechanism did select indicates in her contribution table that she will contribute 1 token if the other three group members contribute on average 18 tokens, then the total contribution of the group to the project is given by 16 + 18 + 20 + 1 = 55 tokens. Each group member will therefore earn 0.4 × 55 = 22 Guilders from the project plus their respective income from their own private account. If, instead, the randomly selected group member indicates in

her contribution table that she contributes 19 if the others contribute on average 18 tokens, then the total contribution of the group to the project is 16 + 18 + 20 + 19 = 73 tokens. Each group member would therefore earn 0.4 × 73 = 29.2 Guilders from the project plus their respective income from their own private account.

The random mechanism

Each group member is assigned a Group Member ID between 1 and 4, which denotes this participant's number inside her group. Moreover, participant number 2 was randomly selected at the very beginning of the experiment. This participant will draw a ball from an urn after all participants have made their unconditional contribution and have filled out their contribution table. Each ball in the urn has a different colour and each colour corresponds to a Group Member ID: orange = 1, blue = 2, yellow = 3, green = 4. The resulting number will be entered into the computer. If your Group Member ID is drawn, then your contribution table will determine your contribution to the project. For all other members of your group, the unconditional contributions will be relevant. Otherwise, your unconditional contribution determines your contribution.

If you have any questions, please raise your hand and a member of the experiment team will come and answer them in private.

Study 1: Without Punishment treatment – Part 2

You are now taking part in a second experiment. Your payoff from this experiment is completely unrelated to the decisions you have made in the previous one. The money you earn in this experiment will be added to what you earned in the first experiment. As before, the Guilders you have earned will be converted to Pounds at the following rate:

1 Guilder = 0.20 Pounds

As in the previous experiment, all participants will be randomly divided into groups of four. However, the composition of the group is entirely new. **You will not learn who the other people in your group are at any point.**

The decision situation

The decision situation is the same as the one described on the first instruction sheet: Each participant receives an endowment of **20 tokens**. You have to decide how many of these 20 tokens you contribute to a group project and how many you keep for yourself. The three other

members of your group have to make the same decision. However, this time you will make only an unconditional contribution to the project. There will be no contribution table.

Like in the first experiment, you will make the contribution decision in the second experiment **only once**.

You will see the following screen when making your contribution decision:

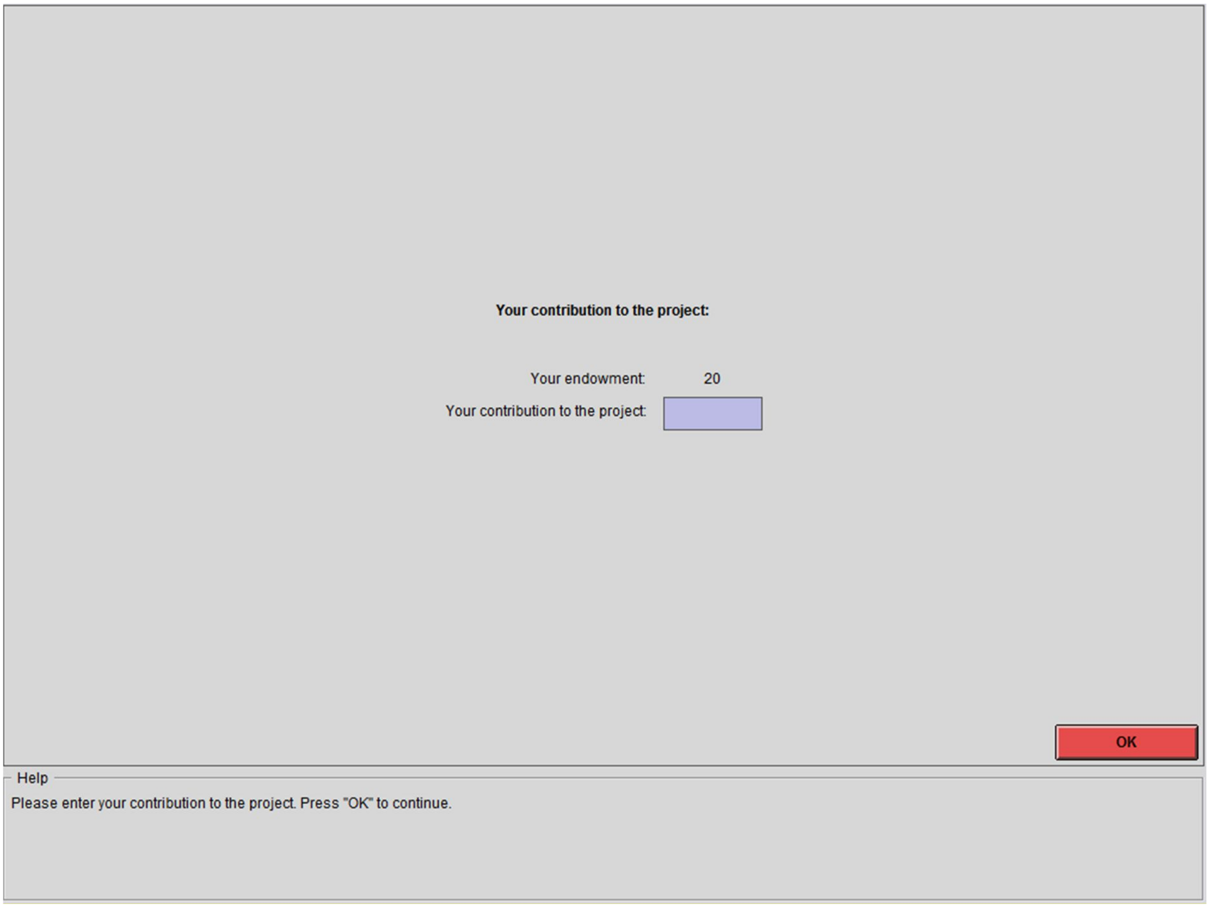

After the contribution decision, you will see how many tokens each of the other three group members has contributed to the project and their corresponding income from this contribution decision. Nonetheless, the identities of your group members will not be revealed at any time.

The payoffs

The calculation of payoffs is identical to the previous experiment. The income of every member of the group is calculated in the same way. Your income consists of two components:

(1) The first component is the amount of tokens that you keep for yourself. Every token that you do not contribute to the project automatically belongs to you and earns you one Guilder.

(2) The second component is your personal return from the group project. For all of the tokens contributed to the project the following happens: the project's value will be multiplied by 1.6 and this amount will be divided equally among all four members of the group.

For example, if 1 token is contributed to the project, the project's value increases to 1.6 Guilders. This amount is divided equally among all four members of the group. Thus every group member receives 0.4 Guilders.

The following function is exactly the same as in the previous experiment and illustrates your income in Guilders:

$$\textit{Your Total Income} = 20 - \textit{Your Contribution} + 0.4 \times (\textit{Group Project})$$

If you have any questions, please raise your hand and a member of the experiment team will come and answer them in private.

Study 1: With Punishment treatment – Part 1

Same as in Part 1 of the Without Punishment treatment.

Study 1: With Punishment treatment – Part 2

You are now taking part in a second experiment. Your payoff from this experiment is completely unrelated to the decisions you have made in the previous one. The money you earn in this experiment will be added to what you earned in the first experiment. As before the Guilders you have earned will be converted to Pounds at the following rate:

1 Guilder = 0.20 Pounds

As in the previous experiment, all participants will be randomly divided into groups of four. However, the composition of the group is entirely new. **You will not learn who the other people in your group are at any point.**

The decision situation

The decision situation is the same as the one described on the first instruction sheet: Each participant receives an endowment of **20 tokens**. You have to decide how many of these 20 tokens you contribute to a group project and how many you keep for yourself. The three other

members of your group have to make the same decision. However, this time you will make only an unconditional contribution to the project. There will be no contribution table.

After the contribution decision, there will be a **second stage**. At this stage, you will see how many tokens each of the other three group members has contributed to the project and their corresponding income from this contribution decision. Nonetheless, the identities of your group members will not be revealed at any stage. You can either **decrease** or **leave unchanged** the income of each other group member by assigning **deduction points** to them. The other group members can also decrease your income, by allocating deduction points to you, if they wish to do so.

Deduction points

In stage 2, you can assign **between 0 and 5 deduction points to each other group member.** The maximum number of deduction points, you can allocate to the other group members together is therefore 15 deduction points.

For each deduction point that you assign, there is a cost to you of one Guilder. Thus, the total cost to you in Guilders of assigning deduction points to other group members is given by the total number of deduction points that you assign.

For each deduction point that you assign to a particular group member, you will decrease their income by 2 Guilders unless their income is already exhausted. For example, if you give a group member 2 deduction points, you will decrease this group member's income by 4 Guilders.

Your **own income will be reduced by 2 Guilders for each deduction point that is assigned to you** by the other three group members. If all of your income from the first stage of this experiment is exhausted, it cannot be reduced any further by other group members.

You will see the following screen at stage 2:

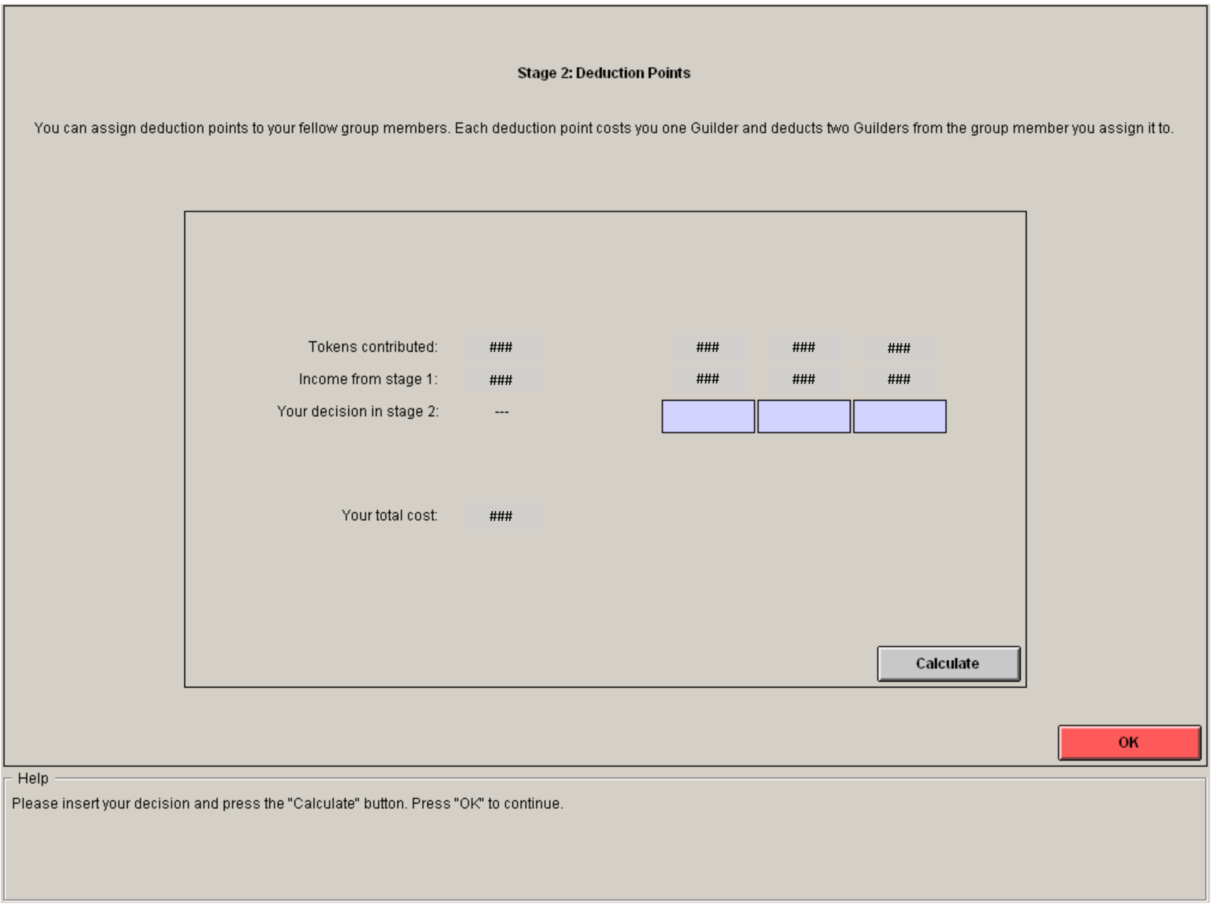

The column on the left shows your contribution and your income from the first stage. The other three columns indicate the contribution of your group members and their income from the first stage.

If you do not wish to change the income of the other group members, type "*0*" into the fields next to *"Your decision in stage 2"*. In case you want to assign deduction points, enter the number of deduction points you want to assign into this field. You must enter a decision into every field and press the "*Calculate"* button. This will display the cost of your decision. Until you press the "*OK*" button, you can still change your decision. To recalculate the costs after making a change, simply press the "*Calculate*" button again.

The payoffs

Your total income in Guilders from the two stages will be calculated as follows:

Your Income From Stage 1 = 20 − *Your Contribution* + 0.4 × (*Group Project*)

Total Income After Stage 2 = *Income From Stage 1* **(1)**

− 2 × (*Sum Of Deduction Points Assigned To You*) **(2)**

− (*Deduction Points Assigned By You*)

if (1) + (2) is greater or equal to 0.

Total Income After Stage 2 = 0 − (Deduction Points Assigned By You)

if (1) + (2) is less than 0.

Please note that your income in Guilders after stage 2 can be negative, if the cost of deduction points assigned by you exceeds your income from stage 1 less any reduction in your income caused by other group members.

However, at the end of the experiment and in addition to the calculation just given, you and the other members of your group will each receive a lump sum payment of **10 Guilders.** This payment is to cover losses that you could incur.

If you have any questions, please raise your hand and a member of the experiment team will come and answer them in private.

Study 2: Part 1

Same as in Study 1.

Study 2: Part 2

You are now taking part in a second experiment. Your payoff from this experiment is completely unrelated to the decisions you have made in the previous one. The money you earn in this experiment will be added to what you earned in the first experiment. As before the Guilders you have earned will be converted to Pounds at the following rate:

1 Guilder = 0.20 Pounds

As in the previous experiment, all participants will be randomly divided into groups of four. However, the composition of the group is entirely new. **You will not learn who the other people in your group are at any point.**

The decision situation

The decision situation is the same as the one described on the first instruction sheet: Each participant receives an endowment of **20 tokens**. You have to decide how many of these 20 tokens you contribute to a group project and how many you keep for yourself. The three other members of your group have to make the same decision. However, this time you will make only an unconditional contribution to the project. There will be no contribution table.

After the contribution decision, there will be a **second stage**. At this stage, you will see how many tokens each of the other three group members has contributed to the project and their corresponding income from this contribution decision. Nonetheless, the identities of your group members will not be revealed at any stage. You can either **decrease** or **leave unchanged** the income of each other group member by assigning **deduction points** to them. The other group members can also decrease your income, by allocating deduction points to you, if they wish to do so.

Deduction points

In stage 2, you can assign **between 0 and 5 deduction points to each other group member.** The maximum number of deduction points, you can allocate to the other group members together is therefore 15 deduction points.

For each deduction point that you assign, there is a cost to you of one Guilder. Thus, the total cost to you in Guilders of assigning deduction points to other group members is given by the total number of deduction points that you assign.

For each deduction point that you assign to a particular group member, you will decrease their income.

Before allocating deduction points, the computer will randomly draw your deduction factor, which will be either 1 or 3. The magnitude of **income reduction from each deduction point you allocate is determined by this deduction factor.** If the deduction factor is 1, then each deduction point you allocate to a group member will reduce this group member’s income by 1 Guilder. If the deduction factor is 3, then each deduction point you allocate to a group member will reduce this group member’s income by 3 Guilders. There is a 50% chance of drawing 1

and a 50% chance of drawing 3. The computer will randomly draw the deduction factors of your group members, in exactly the same way. The draws are independent for each participant.

Your **own income can be reduced by the other three group members**. The income reduction depends on the number of deduction points you receive and the respective deduction factor of the other group members. If all of your income from the first stage of this experiment is exhausted, it cannot be reduced any further by other group members.

At stage 2, you will learn your deduction factor and then see the following screen:

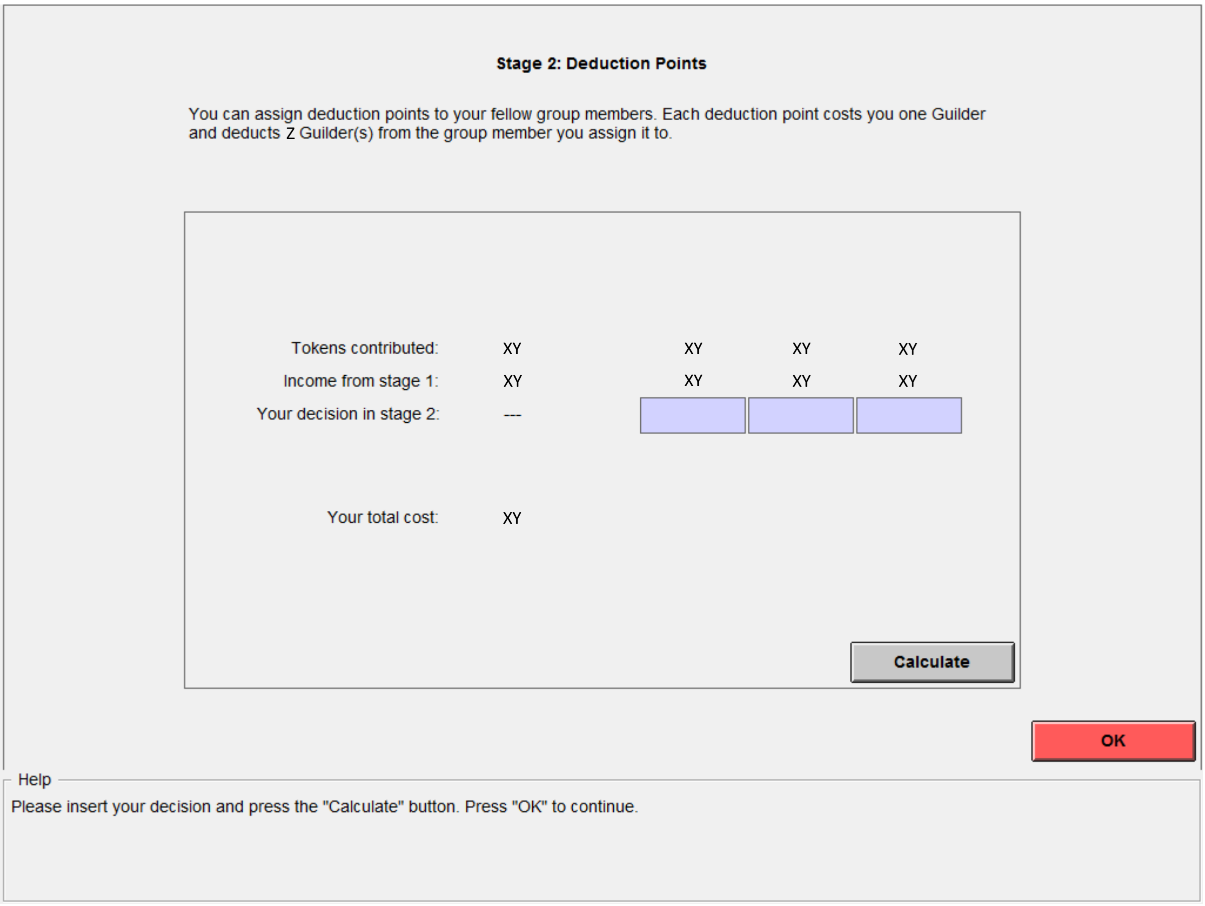

The column on the left shows your contribution and your income from the first stage. The other three columns indicate the contribution of your group members and their income from the first stage.

If you do not wish to change the income of the other group members, type "*0*" into the fields next to *"Your decision in stage 2"*. In case you want to assign deduction points, enter the number of deduction points you want to assign into this field. You must enter a decision into every field and press the "*Calculate*" button. This will display the cost of your decision. Until

you press the "*OK*" button, you can still change your decision. To recalculate the costs after making a change, simply press the "*Calculate*" button again.

The payoffs

Your total income in Guilders from the two stages will be calculated as follows (we will refer to your three group members as A, B and C):

Your Income From Stage 1 = 20 − *Your Contribution* + 0.4 × (*Group Project*)

Total Income After Stage 2 = *Income From Stage 1* **(1)**

− (*Deduction Points Assigned To You By A*) × *A's deduction factor*
− (*Deduction Points Assigned To You By B*) × *B's deduction factor* **(2)**
− (*Deduction Points Assigned To You By C*) × *C's deduction factor*

− (*Deduction Points Assigned By You*)

if (1) + (2) is greater or equal to 0.

Total Income After Stage 2 = 0 − (Deduction Points Assigned By You)

if (1) + (2) is less than 0.

Please note that your income in Guilders after stage 2 can be negative, if the cost of deduction points assigned by you exceeds your income from stage 1 less any reduction in your income caused by other group members.

However, at the end of the experiment and in addition to the calculation just given, you and the other members of your group will each receive a lump sum payment of **10 Guilders.** This payment is to cover losses that you could incur.

If you have any questions, please raise your hand and a member of the experiment team will come and answer them in private.

Emotions elicitation in Study 1 and 2

The screen below shows the instructions for the emotions elicitation procedure used in the second phase of Study 1 and 2. Subjects were informed about their group member's respective contribution decisions and reported their emotional responses on the ranking scales.

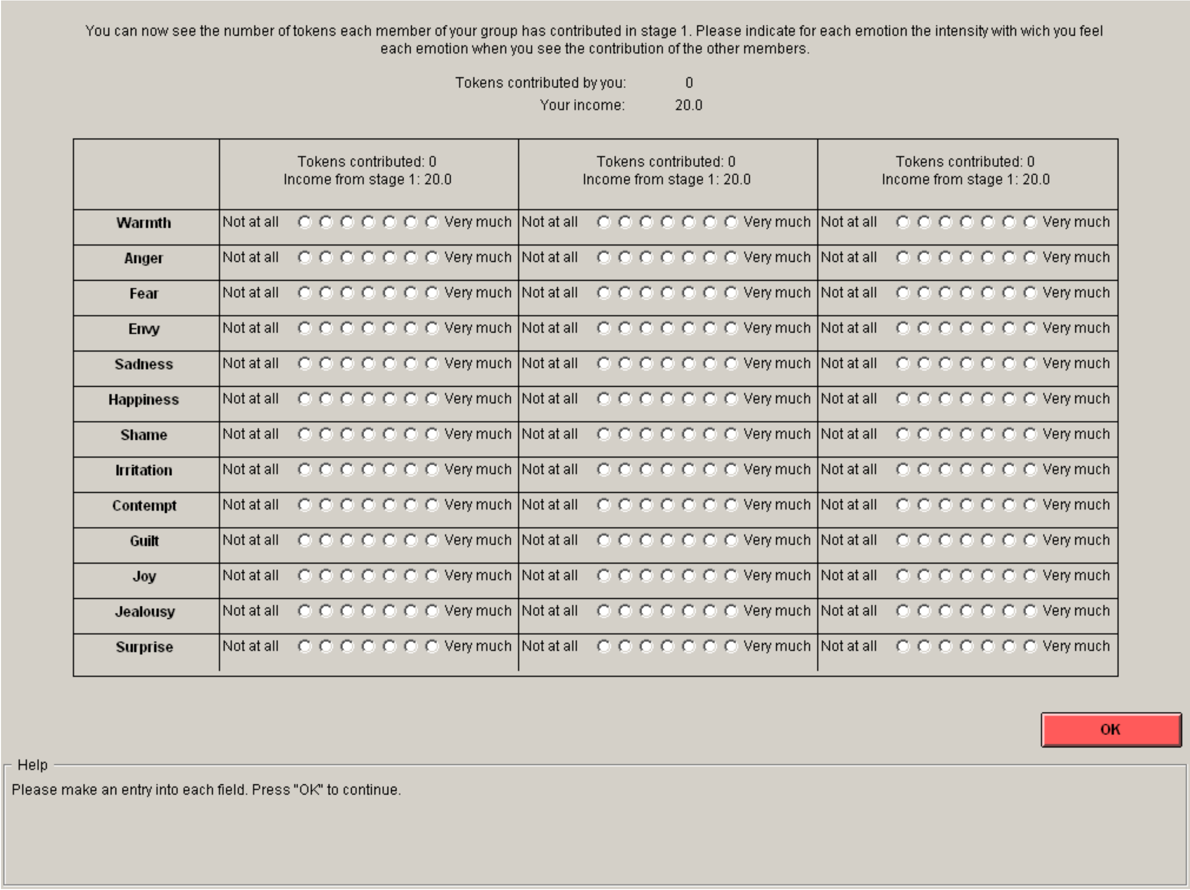

You can now see the number of tokens each member of your group has contributed in stage 1. Please indicate for each emotion the intensity with wich you feel each emotion when you see the contribution of the other members.

Tokens contributed by you: 0
Your income: 20.0

	Tokens contributed: 0 Income from stage 1: 20.0	Tokens contributed: 0 Income from stage 1: 20.0	Tokens contributed: 0 Income from stage 1: 20.0
**Warmth**	Not at all ○ ○ ○ ○ ○ ○ ○ Very much	Not at all ○ ○ ○ ○ ○ ○ ○ Very much	Not at all ○ ○ ○ ○ ○ ○ ○ Very much
**Anger**	Not at all ○ ○ ○ ○ ○ ○ ○ Very much	Not at all ○ ○ ○ ○ ○ ○ ○ Very much	Not at all ○ ○ ○ ○ ○ ○ ○ Very much
**Fear**	Not at all ○ ○ ○ ○ ○ ○ ○ Very much	Not at all ○ ○ ○ ○ ○ ○ ○ Very much	Not at all ○ ○ ○ ○ ○ ○ ○ Very much
**Envy**	Not at all ○ ○ ○ ○ ○ ○ ○ Very much	Not at all ○ ○ ○ ○ ○ ○ ○ Very much	Not at all ○ ○ ○ ○ ○ ○ ○ Very much
**Sadness**	Not at all ○ ○ ○ ○ ○ ○ ○ Very much	Not at all ○ ○ ○ ○ ○ ○ ○ Very much	Not at all ○ ○ ○ ○ ○ ○ ○ Very much
**Happiness**	Not at all ○ ○ ○ ○ ○ ○ ○ Very much	Not at all ○ ○ ○ ○ ○ ○ ○ Very much	Not at all ○ ○ ○ ○ ○ ○ ○ Very much
**Shame**	Not at all ○ ○ ○ ○ ○ ○ ○ Very much	Not at all ○ ○ ○ ○ ○ ○ ○ Very much	Not at all ○ ○ ○ ○ ○ ○ ○ Very much
**Irritation**	Not at all ○ ○ ○ ○ ○ ○ ○ Very much	Not at all ○ ○ ○ ○ ○ ○ ○ Very much	Not at all ○ ○ ○ ○ ○ ○ ○ Very much
**Contempt**	Not at all ○ ○ ○ ○ ○ ○ ○ Very much	Not at all ○ ○ ○ ○ ○ ○ ○ Very much	Not at all ○ ○ ○ ○ ○ ○ ○ Very much
**Guilt**	Not at all ○ ○ ○ ○ ○ ○ ○ Very much	Not at all ○ ○ ○ ○ ○ ○ ○ Very much	Not at all ○ ○ ○ ○ ○ ○ ○ Very much
**Joy**	Not at all ○ ○ ○ ○ ○ ○ ○ Very much	Not at all ○ ○ ○ ○ ○ ○ ○ Very much	Not at all ○ ○ ○ ○ ○ ○ ○ Very much
**Jealousy**	Not at all ○ ○ ○ ○ ○ ○ ○ Very much	Not at all ○ ○ ○ ○ ○ ○ ○ Very much	Not at all ○ ○ ○ ○ ○ ○ ○ Very much
**Surprise**	Not at all ○ ○ ○ ○ ○ ○ ○ Very much	Not at all ○ ○ ○ ○ ○ ○ ○ Very much	Not at all ○ ○ ○ ○ ○ ○ ○ Very much

OK

Help
Please make an entry into each field. Press "OK" to continue.

Supplementary References

1. Fischbacher, U., Gächter, S. & Fehr, E. Are people conditionally cooperative? Evidence from a public goods experiment. *Econ. Lett.* **71**, 397-404, doi:10.1016/S0165-1765(01)00394-9 (2001).
2. Fischbacher, U., Gächter, S. & Quercia, S. The behavioral validity of the strategy method in public good experiments. *J. Econ. Psychol.* **33**, 897-913, doi:10.1016/j.joep.2012.04.002 (2012).
3. Fischbacher, U. & Gächter, S. Social preferences, beliefs, and the dynamics of free riding in public goods experiments. *Am. Econ. Rev.* **100**, 541-556, doi:10.1257/aer.100.1.541 (2010).
4. Herrmann, B. & Thöni, C. Measuring conditional cooperation: a replication study in Russia. *Exp. Econ.* **12**, 87-92, doi:10.1007/s10683-008-9197-1 (2009).
5. Kocher, M. G., Cherry, T., Kroll, S., Netzer, R. J. & Sutter, M. Conditional cooperation on three continents. *Econ. Lett.* **101**, 175-178, doi:10.1016/j.econlet.2008.07.015 (2008).

6. Martinsson, P., Nam, P. K. & Villegas-Palacio, C. Conditional cooperation and disclosure in developing countries. *J. Econ. Psychol.* **34**, 148-155, doi:10.1016/j.joep.2012.09.005 (2013).
7. Volk, S., Thöni, C. & Ruigrok, W. Temporal stability and psychological foundations of cooperation preferences. *J. Econ. Behav. Organ.* **81**, 664-676, doi:10.1016/j.jebo.2011.10.006 (2012).
8. Burton-Chellew, M. N., El Mouden, C. & West, S. A. Conditional cooperation and confusion in public-goods experiments. *Proc. Natl Acad. Sci.* **113**, 1291-1296, doi:10.1073/pnas.1509740113 (2016).
9. Burlando, R. M. & Guala, F. Heterogeneous agents in public goods experiments. *Exp. Econ.* **8**, 35-54, doi:10.1007/s10683-005-0436-4 (2005).
10. Kurzban, R. & Houser, D. Experiments investigating cooperative types in humans: a complement to evolutionary theory and simulations. *Proc. Natl Acad. Sci.* **102**, 1803-1807, doi:10.1073/pnas.0408759102 (2005).
11. Fallucchi, F., Luccasen, R. A. & Turocy, T. L. Identifying discrete behavioural types: a re-analysis of public goods game contributions by hierarchical clustering. CBESS Discussion Paper 17-01R (2017).
12. Fosgaard, T. R., Hansen, L. G. & Wengstrom, E. Framing and misperception in public good experiments. *Scand. J. Econ.* **119**, 435-456, doi:10.1111/sjoe.12165 (2017).
13. Herrmann, B., Thöni, C. & Gächter, S. Antisocial punishment across societies. *Science* **319**, 1362-1367, doi:10.1126/science.1153808 (2008).
14. Nikiforakis, N. Punishment and counter-punishment in public good games: can we really govern ourselves? *J. Public. Econ.* **92**, 91-112, doi:10.1016/j.jpubeco.2007.04.008 (2008).
15. Cubitt, R. P., Drouvelis, M. & Gächter, S. Framing and free riding: emotional responses and punishment in social dilemma games. *Exp. Econ.* **14**, 254-272, doi:10.1007/s10683-010-9266-0 (2011).
